# Ethnobotany, Phytochemistry and Pharmacological Effects of Plants in Genus *Cynanchum* Linn. (Asclepiadaceae)

**DOI:** 10.3390/molecules23051194

**Published:** 2018-05-16

**Authors:** Lu Han, Xiuping Zhou, Mengmeng Yang, Li Zhou, Xinxin Deng, Shijie Wei, Wenping Wang, Zhizhong Wang, Xue Qiao, Changcai Bai

**Affiliations:** 1 Key Laboratory of Hui Ethnic Medicine Modernization, Ministry of Education, Ningxia Medical University, Yinchuan 750004, China; lulu2008han@163.com (L.H.); 18295498705@163.com (X.Z.); 15809605110@163.com (M.Y.); zhouli1028@163.com (L.Z.); dengxx1012@163.com (X.D.); weishijie818@sohu.com (S.W.); wpwang2015@163.com (W.W.); pxwzz@163.com (Z.W.); snow_36man@126.com (X.Q.); 2 Ningxia Research Center of Modern Hui Medicine Engineering and Technology; Yinchuan 750004, China

**Keywords:** *Cynanchum* L., ethnobotany, phytochemistry, pharmacological effects, review

## Abstract

Genus *Cynanchum* L. belongs to the family Asclepiadaceae, which comprise more than 200 species distributed worldwide. In Chinese medical practice, numerous drugs (such as tablets and powders) containing different parts of plants of this genus are used to treat snake bites, bruises, osteoblasts, rheumatoid arthritis and tumors. A search for original articles published on the cynanchum genus was performed by using several resources, including Flora of China Official Website and various scientific databases, such as PubMed, SciFinder, the Web of Science, Science Direct, and China Knowledge Resource Integrated (CNKI). Advances in the botanical, ethnomedicinal, phytochemical, and pharmacological studies of this genus are reviewed in this paper. Results showed that more than 440 compounds, including C21 steroids, steroidal saponins, alkaloids, flavonoids and terpene, have been isolated and identified from *Cynanchum* plants up to now. In vivo and in vitro studies have shown that plants possess an array of biological activities, including anti-tumor, neuroprotective and anti-fungal effects. Popular traditional prescription of *Cynanchum* sp. was also summed up in this paper. However, many *Cynanchum* species have received little or no attention. Moreover, few reports on the clinical use and toxic effects of *Cynanchum* sp. are available. Further attention should be focused on the study of these species to gather information on their respective toxicology data and relevant quality-control measures and clinical value of the crude extracts, active compounds, and bioactive metabolites from this genus. Further research on *Cynanchum* sp. should be conducted, and bioactivity-guided isolation strategies should be emphasized. In addition, systematic studies of the chemical composition of plants should be enhanced.

## 1. Introduction

*Cynanchum* L. is a large genus in the Asclepiadaceae family comprising approximately 200 species. Many of these plants have been used for a long time in traditional Chinese medicine (TCM) for the treatment of common and chronic diseases. Plants of this genus are distributed worldwide, including in East Africa, the Mediterranean region, the tropical zone of Europe, and the subtropical and temperate zones of Asia [1]. A total of 53 species and 12 varieties are native to the southwestern region of China [2]. However, only 33 species of the genus *Cynanchum* have been systematically studied to date [3].

*Cynanchum* L. is an important taxonomic group in the Asclepiadaceae family because numerous species of this genus have several application prospects other than in the field of medicine. These species of *Cynanchum* include *C*. *sibiricum*, *C*. *chinense*, *C*. *auriculatum*, *C*. *officinale*, *C*. *bungei*, *C*. *otophyllum*, *C*. *corymbosum*, *C*. *amplexicaule*, *C*. *forrestii*, *C*. *stauntonii*, *C*. *vincetoxicum*, *C*. *inamoenum*, *C*. *atratum* (CA), *C*. *glaucescens*, *C*. *paniculatum*, *C*. *komarovii*, *C*. *versicolor*, *C*. *chekiangense* and *C*. *mooreanum* (http://frps.eflora.cn/frps/Cynanchum). These plants are traditionally used to treat snake bites, bruises, osteoblasts, rheumatoid arthritis and tumors. Some plants are poisonous; thus, they are used to kill agricultural pests and tigers because of their higher toxicity than other plants [1]. In addition, modern pharmacological studies showed that *Cynanchum* plants exert significant immune regulation, anti-oxidation, anti-tumor and other pharmacological effects [4].

Given the high medicinal value of anti-tumor, immune regulation and anti-oxidation of *Cynanchum*, a growing number of studies have been carried out on the chemical composition of the genus [5]. At present, 450 compounds from *Cynanchum* sp. have been isolated. Results showed that C21 steroids are the main chemical constituents of this genus, as well as acetophenones, alkaloids and certain alkyd compounds.

For our literature review, we systematically summarized the resources, folk application, chemical composition and pharmacological activity of *Cynanchum*, and proposed certain suggestions according to its research status to provide reference for the comprehensive development and sustainable utilization of the species in this genus.

## 2. Ethnomedicinal Uses

According to our review on the monographs and literature, 17 medicinal plants are included in genus *Cynanchum*; which are *C*. *sibiricum*, *C*. *chinense*, *C*. *auriculatum*, *C*. *officinale*, *C*. *bungei*, *C*. *otophyllum*, *C*. *corymbosum*, *C*. *amplexicaule*, *C*. *forrestii*, *C*. *stauntonii*, *C*. *vincetoxicum*, *C*. *inamoenum*, CA, *C*. *glaucescens*, *C*. *paniculatum*, *C*. *komarovii*, *C*. *versicolor*, *C*. *chekiangense* and *C*. *mooreanum*. In China, plants of genus *Cynanchum* are mainly distributed in the southwest, northwest and northeast provinces. In local medicine, some plant roots have been used to clear away heat evil and expel superficial evils, eliminate stasis, activate blood circulation, induce diuresis and reduce edema. This review summarizes local using of *Cynanchum* plants in the national medicine, as shown in Table 1.

In addition, compound medication has always been an important feature of folk medicine. *Cynanchum* plants and other Chinese herbs are used in a number of prescriptions, such as Baiweiwan and Baiweisan. *Cynanchum* plants also present a long history as a folk medicine, thus providing an important reference for clinical practice (Table 2).

## 3. Chemical Constituents

**Figure 1:**
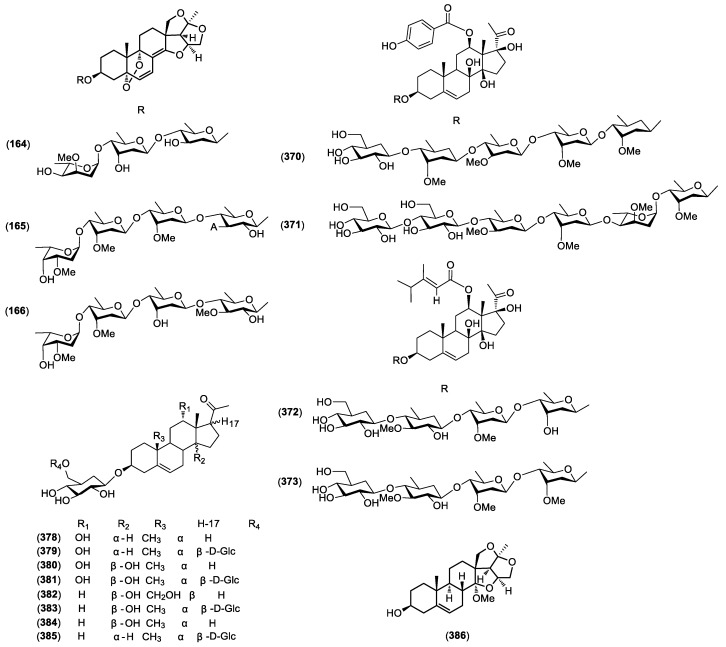
Structures of newly isolated C21 steroid compounds from *Cynanchum* species in 2016–2017.

At present, more than 400 compounds have been isolated from genus *Cynanchum*. These compounds include 388 steroids, 30 benzenes and its derivatives, 13 alkaloids, 10 flavonoids, 9 terpenes and other compounds (Table 3). The chemical structures of the primary compounds are shown in Figure 1.

### 3.1. C21 Steroids

The C21 steroid compounds all have the basic skeleton of pregnane, which containing 21 carbon atoms or a derivative of its isomers. C21 steroid constituents in *Cynanchum* sp. can be classified into two groups on the basis of their carbon frameworks as typical and modified C21 steroids. According to the different pregnane skeletons, these compounds can be finally divided into the following five types: the normal four-ring pregnane type, 14,15-secopregnanetype, 13,14:14,15-diseco-pregnane type, aberrant 14,15-seco-pregnane type and 12,13-seco-14,18-nor-pregnane type. In C21 steroidal glycosides, sugar moiety is linked most frequently at C-3 to a hydroxyl group of the pregnane aglycone, which contains one to seven sugar units with mode of 1→4, and is generally composed of a linear (rather than a branched) oligosaccharide chain. The most common sugar residues are hexose (glucose), 6-deoxyhexose (thevetose) and 2,6-dideoxyhexoses (cymarose, oleandrose, digitoxose, diginose, sarmentose and canarose). In 2016, Gu et al. on the C21 steroid have been comprehensively and fully explained [2]. Therefore, we summarized the newly isolated compounds from *Cynanchum* sp. in 2016–2017 (Figure 1).

### 3.2. Benzene and Its Derivatives

**Figure 2:**
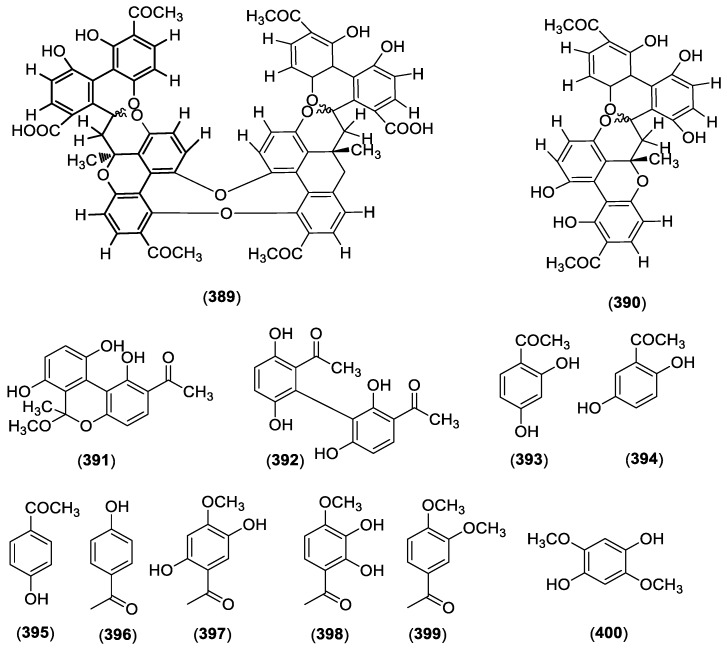

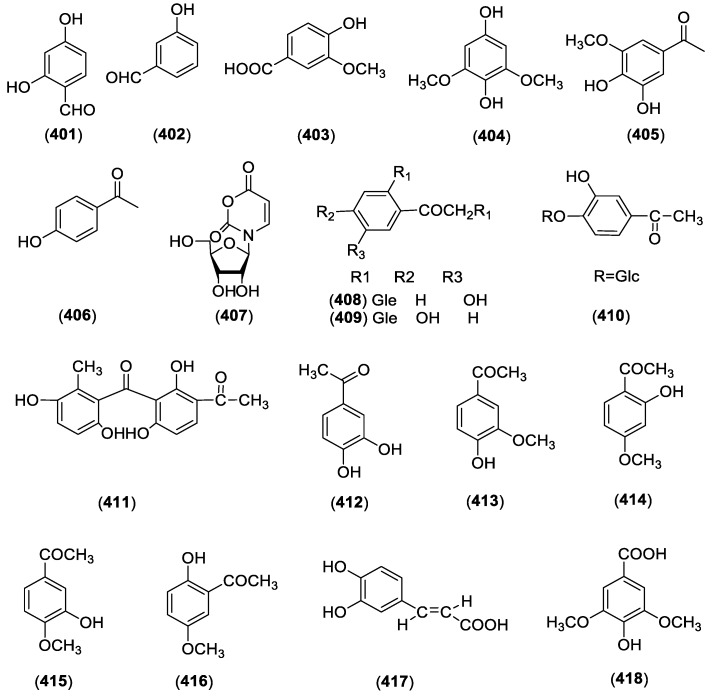
Structures of compounds **389**–**418** from *Cynanchum* species.

Benzene and its derivatives are also found in *Cynanchum* plants. These components are mainly acetophenone derivatives, and most of them were isolated from *C*. *paniculatum*, *C*. auriculatum and *C*. *stauntonii*. The acetophenones in *Cynanchum* sp. include cynantetrone (**389**), cynantetrone A (**390**), cynandione A (**391**), cynandione B (**392**) [78], 2,4-dihydroxyacetophenone (**393**), 2,5-dihydroxyacetophenone (**394**) [79], 4-hydroxyacetophenone (**395**) [25], 4-acetylphenol (**396**), 2,5-dihydroxy-4-methoxyacetophenone (**397**), 2,3-dihydroxy-4-methoxyacetophenone (**398**) [81], acetoveratrone (**399**), 2,5-dimethoxyhydroquinone (**400**), resacetophenone (**401**), *m*-acetylphenol (**402**), vanillic acid (**403**), 3,5-dimethoxyhydroquinone (**404**) [80], acetovanillone (**405**), *p*-hydroxyacetophenone (**406**), 3-(*β*-d-ribofuranosyl)-2,3-dihydro-6H-1,3-oxazine-2,6-dione (**407**), bungeiside A (**408**), cynanoneside B (**409**) [3], cynanoneside A (**410**) [82], baishouwubenzophenone (**411**) [83], 3,4-dihydroxyacetophenone (**412**) [39], 4′-hydroxy-3′-methoxyacetophenone (**413**) [84], paeonol (**414**), isopaeonol (**415**), 2-hydroxy-5-methoxyacetophenone (**416**) [86], caffeic acid (**417**) [85] and syringic acid (**418**) [25]. Structures of these compounds are shown in Figure 2.

### 3.3. Alkaloids

**Figure 3:**
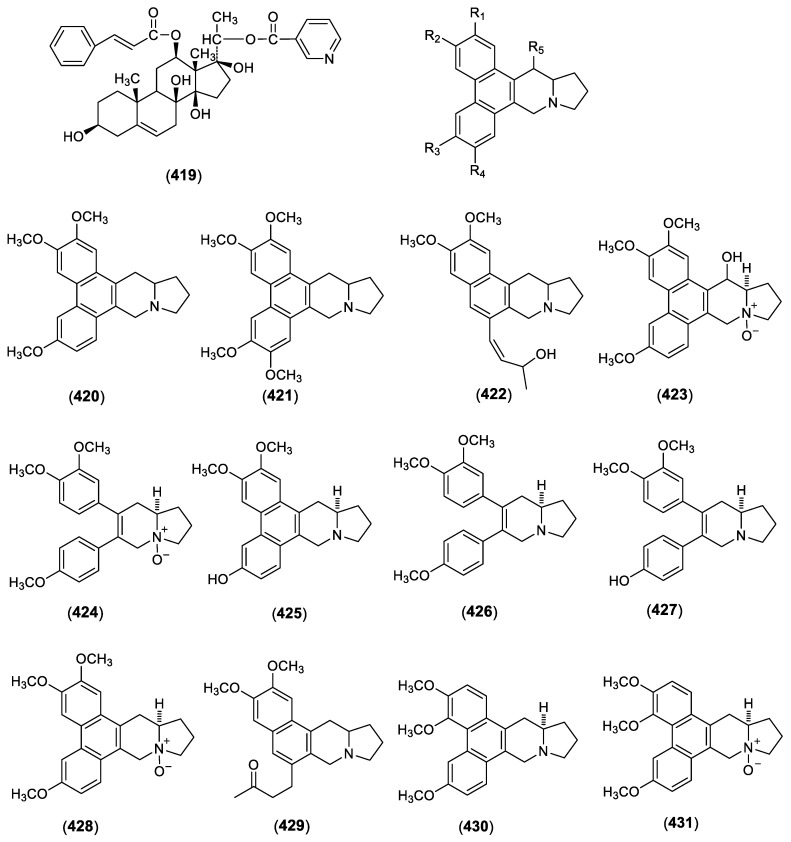
Structures of compounds **419**–**431** from *Cynanchum* species.

Studies showed that alkaloids are only found in several plants of genus Cynanchum, and some of these alkaloids showed notable bioactivity. To date, 13 alkaloids were identified from genus *Cynanchum*. These alkaloids include a steroidal alkaloid gagaminine (**419**) [94] and fourteen phenanthroindolizidine alkaloids. The phenanthroindolizidine is an alkaloid with a basic skeleton that is a pentacyclic structure with a phenanthrene ring and a indolizidine ring, in which the phenanthrene ring contains a plurality of methoxy groups or hydroxyl groups, and some of the alkaloids also contain a methyl group or a hydroxyl group on the indolizidine ring. In this type of alkaloid, the phenanthrene ring of some compounds is not formed, and some compounds are nitrogen oxides. In addition to compound **419**, compounds **420**–**432** have been identified as phenanthroindolizidine alkaloids. These compounds were isolated from aerial parts of *C. vincetoxicum* and identified as antofine (**420**), tylophorine (**421**), vincetene (**422**) [88], (-)-10*β*, 13a*α*-14*β*-hydroxyantofine *N*-oxide (**423**), (-)-10-*β*, 13a*α*-secoantofine *N*-oxide (**424**) [90], (−)-(*R*)-13a*α*-6-*O*-desmethylantofine (**425**), (−)-(*R*)-13a*α*-secoantofine (**426**), (−)-(*R*)-13a*α*-6-*O*-desmethylsecoantofine (**427**) [91], (-)-10*β*-antofine *N*-oxide (**428**) [90], 2,3-dimethoxy-6-(3-oxo-butyl)-7,9,10,11,11a,12-hexahydrobenzo[*f*]pyrrolo[*1*,*2-b*]isoquinoline (**429**), 7-demethoxytylophorine (**430**) and 7-demethoxy-tylophorine *N*-oxide (**431**) [92]. Structures of these compounds are shown in Figure 3. 

### 3.4. Flavones

**Figure 4:**
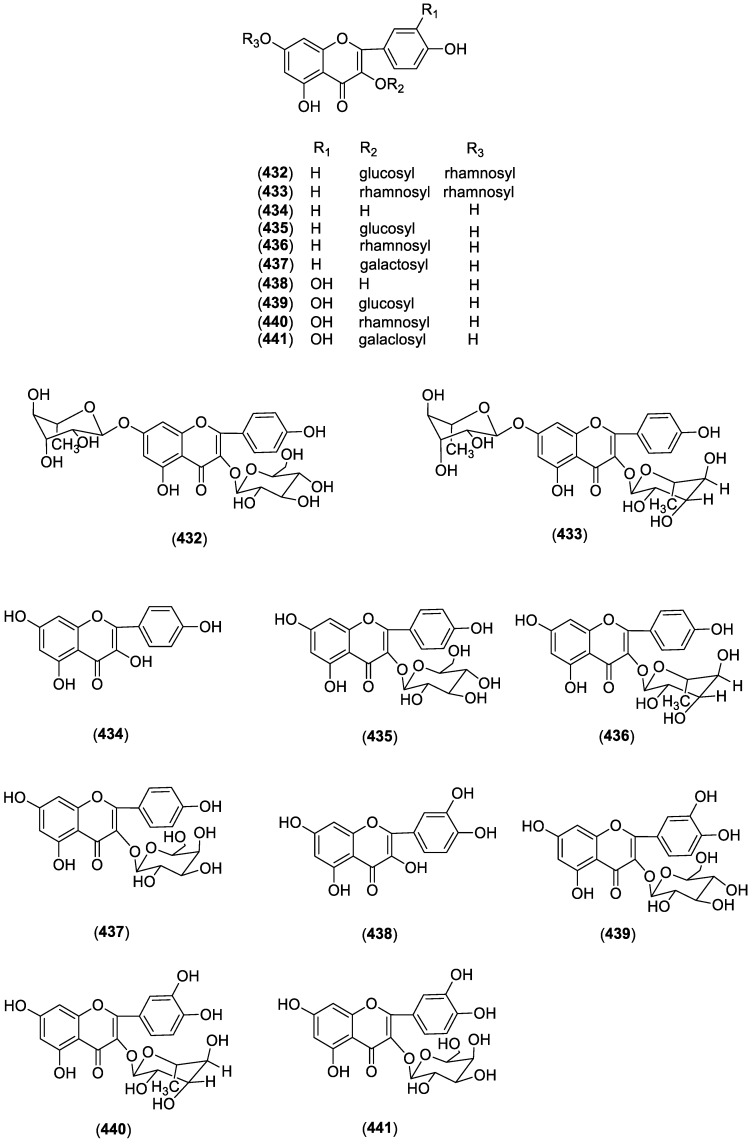
Structures of compounds **432**–**441** from *Cynanchum* species.

To date, there are few flavonoids isolated and identified from genus Cynanchum and most of them are flavonoid glycosides with 3- or 7-linked glycans. 7-*O*-*α*-l-rhamnopyranosyl-kaempferol-3-*O*-*β*-d-glucopyranoside (**432**) and 7-*O*-*α*-l-rhamnopyranosyl-kaempferol-3-*O*-*α*-l-rhamnopyranoside (**433**) were identified from *C*. *chinense* [93]. Eight flavone components kaempferol (**434**), astragalin (**435**), afzelin (**436**), trifolin (**437**), quercetin (**438**), isoquercitrin (**439**), quercitrin (**440**) and hyperin (**441**) [85] were isolated from the aerial part of *C*. *taiwanianum*. Structures of these compounds are shown in Figure 4. 

### 3.5. Terpene

**Figure 5:**
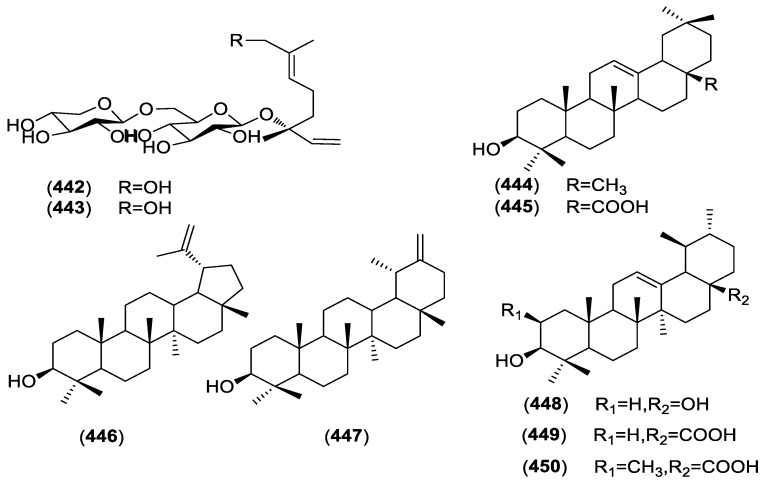
Structures of compounds **442**–**450** from *Cynanchum* species.

The basic skeleton of terpenoids is a type of compound composed of isoprene structural units linked. There are two monoterpene diglycosides neohancoside A (**442**) and B (**443**) are monoterpene diglycosides isolated from *C*. hancockianum A and B [95]. In addition, there are also seven pentacyclic triterpene compounds *β*-amyrin (**444**), *α*-amyrin (**445**), lupeol (**446**), taraxasterol (**447**), ursolic acid (**448**), oleanolic acid (**449**) and maslinic acid (**450**), were isolated from the roots of *C*. *paniculatum* [86]. The structures of these compounds are shown in Figure 5.

### 3.6. Others

In addition to the above-mentioned main components, other components, such as carboxylic acid, alcohol, ester and lignin, are foundin *Cynanchum*. These compounds include azelaic acid, suberic acid and succinic acid [85]; 3,3′-dimethoxy-4,9,9′-trihydroxy-benzofuranoid ligan-7′-ene-9-*O*-*β*-d-glucoside; 3,5-dihydroxybenzoic acid methyl ester; 4-dydroxybenzoic acid; 2,5-dihydroxybenzoic acid methyl ester [56]; conduritol F [3], *p*-menthane-1,7,8-triol, 1-*p*-menthane-8,9-triol, *p*-menthane-1,8,9-triol,trans-terpin [37], 2,6,2′,6′-tetramethoxy-4,4′-bis(2,3-epoxy-1-hydroxypropyl)-biphenyl [39] and (+)-(7*S*,8*R*,7′*E*)-5-hydroxy-3,5′-dimethoxy-4′,7-epoxy-8,3′-neolign-7′-ene-9,9′-diol 9′-ethyl ether [63].

## 4. Pharmacology

In recent years, research reports on the chemical constituents and pharmacological activities of plants of genus *Cynanchum* have shown an increasing trend. An increasing number of researchers show special interest in this genus and its therapeutic properties in the field of traditional Chinese medicine. In Table 4, it was summarized on the major ethnic pharmacological uses of *Cynanchum* sp. and the status of modern pharmacological evaluation. Its pharmacological effects are mainly anti-cancer, anti-inflammatory, anti-virus, appetite suppressing and other effects.

### 4.1. Anti-Cancer

Crude extracts and compounds have significant activity against tumor cells, such as the SMMC-7721, MCF-7, Hela, K562, SHG44, HCT-8, A549, PC3, PLC/PRF/5, KB, T-24, A549, SK-OV-3, SK-MEL-2, HCT-15, Col2, 212, HepG2 and U251 cell lines in vitro. However, few studies have been conducted on the anti-cancer activity of *Cynanchum* plants in vivo. 

The anti-cancer activity of the ethanol extract of *C*. *auriculatum* and different solvent extraction fractions was studied by inhibiting the growth of sarcoma S180 in mice and In vitro MTT assay. The ethanol extract inhibits K562 cell growth, with the highest inhibition ratio of 24.06% at a concentration of 1 μg/mL [96]. The inhibition rate of petroleum ether to PC3 cells at a concentration of 100 μg/mL is 33.63%. At a concentration of 100 μg/mL, the inhibition ratio of the CHCl_3_ fraction against K562, SHG44, HCT-8, A549 and PC3 are 35.64%, 20.61%, 31.64%, 26.99% and 52.11%, respectively. The inhibitory rates of EtOAc fraction on A549 and PC3 cells are 37.86% and 28.41%, respectively. The *n*-BuOH fraction shows weak cytotoxicity to other cells at the same concentration except for K562 cells. In addition, the ethanol extract and *n*-BuOH fraction inhibit the growth of sarcoma S180 in mice compared with the blank control (*p* < 0.01) at a dose of 100 mg/kg.

Compounds **389** and **392** from the rhizomes of *C*. *taiwanianum* showed significant cytotoxic effects against T-24 cell lines with ED_50_ values of ca. 3.5 and 2.5 mg/mL, respectively. Compound **385** also adversely affected PLC/PRF/5 cell lines (ED_50_ = 2.7 mg/mL) [78].

In 1992, alkaloids **420**–**423** extracted from *C*. *vincetoxicum*, were found to inhibit the growth of MDA-MB-231 mammary carcinoma cells. Compounds **420**, **425**–**429** and **430**, which were isolated from the aerial parts of *C*. *vincetoxicum*, are assessed In vitro using both drug-sensitive KB-3-1 and multidrug-resistant KB-V1 cancer cell lines [90,91]. The results showed that compounds **420**, **425**, **427** and **430** exhibited pronounced cytotoxicity against KB-3-1 and KB-V1 cell lines with IC_50_ (the concentration required for 50% inhibition) values in the low nanomolar range. In addition, Sang et al. found that compound **420**, which is isolated from the root of *C*. *paniculatum*, inhibits the growth of A549 and Col2 cell lines with IC_50_ values of 7.0 ± 0.2 and 8.6 ± 0.3 ng/mL [100]. Ellipticine, as a positive control, exhibited IC_50_ value for A549 and Col2 cancer cells ranging from 300–500 ng/mL. Moreover, Col2 cells considerably accumulate in the G2/M cell cycle when treated with antofine (50 pg/mL) for 48 h. Therefore, this mechanism may be the main process by which antofine inhibits the growth of Col2 cells [100].

Compound **215** was isolated from the roots of *C*. *wilfordii* (CWW) and completely reverse the multidrug resistance of KB-V1 and MCF7/ADR cells to Adriamycin, vinblastine and colchicine at a concentration level of 1 μM [54].The inhibitory ratio of compound **116** isolated from ethyl acetate extract of *C*. *paniculatum* to HL-60 cells at a concentration of 10 μg/mL is 98.14% [35]. Kim et al. evaluated the anti-cancer activity of compounds **232** and **233** isolated from the roots of *C*. *paniculatum* against A549, SK-OV-3, SK-MEL-2 and HCT-15 cell lines In vitro by using the SRB bioassay [58]. Experimental results showed that compounds **232** and **233** have selective cytotoxicity on SK-MEL-2 cells with IC_50_ values of 26.55 and 17.36 μM, respectively.

C21 steroidal compounds, which isolated from genus *Cynanchum* also exhibit strong anti-cancer activity. Compound **120** isolated from the roots of CA showed significant cytotoxic effect against 212 cells, with ED_50_ value of 0.96 μg/mL [39].

Two C21 steroidal glycosides, namely, compounds **175** and **176** that were isolated from the roots of *C*. *auriculatum* are tested on SMMC-7721, MCF-7 and Hela cell lines. The results showed that the IC_50_ values of the two compounds against SMMC-7721 cells are 13.49 and 24.95 μM, respectively. Then, the in vivo assay by using solid tumor model H22 in mice was performed [48]. It was found that compounds **175** and **176** can significantly inhibit the growth of transplantable H22 tumors in mice at doses of 10, 20, and 40 mg/kg compared with positive control 5-FU.

The anti-cancer activities of 17 C21-steroidal pregnane sapogenins, namely, compounds **8**, **167**, **170**–**172**, **174**, **175**, **177**, **200**, **209**–**212**, **221**, **223**, **228** and **417**, were evaluated by activity using HL-60, K-562 and MCF-7 cancer cells [9]. The results suggested that compound **8** shows evident cytotoxicity on HL-60 (IC_50_ = 6.72 μM) and MCF-7 cell lines (IC_50_ = 2.89 μM), whereas compounds **200** and **221** show strong inhibitory activities against K-562 (IC_50_ = 6.72 μM) and MCF-7 cell lines (IC_50_ = 2.49 μM), respectively.

Zhang et al. [46] studied the anti-cancer activity of 26 pregnane glycosides (compounds **37**, **38**, **43**, **168**, **184**–**195**, **204**–**207**, **214**, **220**, **323**, **325**, **368** and **369**) by using three cancer cells (HepG2, Hela and U251). All of these pregnane glycosides compared with the positive compounds 5-FU and cisplatin showed cytotoxic activities (IC_50_ < 100 μM) in varying degrees against these cell lines except compounds **189** and **205** (IC_50_ > 100 μM). Moreover, the cytotoxicity of compounds **38**, **219**, **310**–**317** is evaluated against three human cancer cell lines, that is, HepG2, Hela and U251 [55].

### 4.2. Neuroprotective Effect

With the development of the aging population, the incidence of the neurodegenerative diseases also shows a clear upward trend [118]. Therefore, the mechanisms of prevention and early treatment of these diseases have become one of the focuses of research. Research showed that numerous compounds isolated from genus *Cynanchum* exhibit good neuroprotective effects, thereby indicating its potential for further development.

Compound **391** can protect cultured cortical neurons from toxicity induced by H_2_O_2_, l-glutamate and kainate. Compound **391** showed the most potent neuroprotective activity at a concentration of 50 μM. Given its significant neuroprotective effect on cultured cortical neurons, the compound can effectively protect the neurons from oxidative stress mediated by activating a-amino-3-hydroxy-5-methyl-4-isoxazolepropionate/kainate receptors [104].

The inhibitory activities of compounds **85**–**87** and **278** were tested against acetylcholinesterase (AChE). The result showed that compounds **85** and **86** exhibit the most potent inhibitory activity against AchE, with IC_50_ values of ca.6.4 and 3.6 μM, respectively. Compounds **87** and **278** also show AChE inhibition activity, with IC_50_ values of ca. 52.3 and 152.9 μM, respectively [25]. In addition, the anti-amnesic activity of compound **86** was investigated in passive avoidance and Morris water maze tests [105]. The results showed that compound **86** (1.0 mg/kg body weight i.p.) has significantly ameliorated the memory impairments induced in mice by scopolamine (1.0 mg/kg body weight s.c.).

The neuroprotective effect of compound **398** against glutamate-induced neurotoxicity in mouse hippocampal HT22 cells was investigated; the result revealed that this compound exerts a neuroprotective effect on glutamate-induced neurotoxicity in HT22 cells, with relatively effective protection of 47.55% at 10 μM [81]. In the hippocampal neuronal cell line HT22, compounds **363**, **364** and **322** resist HCA-induced neuronal cell death within a concentration range of 1–30 μM in a concentration-dependent manner [71].

The effects of 19 compounds which have C21 steroidal structure on anti-seizure-like locomotor activity caused by pentylenetetrazole in zebrafish model were also evaluated. The results showed that compounds **30**, **28** and **223** exert a significant therapeutic effect on epilepsy. The results revealed that compound **30** has a therapeutic efficacy of 55% at a concentration of 300 μM, whereas compound 28 shows therapeutic efficacies of 77% and 90% at 100 and 200 μM concentrations, respectively. Meanwhile, compound **223** showed therapeutic efficacies of 65% and 52% at 100 and 200 μM concentrations, respectively. In comparison, the positive control, phenytoin sodium, shows 66% therapeutic efficacy at a concentration of 300 μM. The results also suggested that these three compounds do not exert any nonspecific neurotoxic or sedative effects or affect locomotor activity [16].

In addition, the anti-epileptic activity of 10 C21 steroidal compounds were evaluated by Li et al. by using the mouse maximal electroshock (MES) model after oral administration. The results suggested that five compounds, namely, compounds **326**, **240**, **99**, **96** and **302**, exhibit significant protection activity in a MES-induced mouse seizure model, with ED_50_ values of 48.5, 95.3, 124.1, 72.3 and 88.1 mg/kg, respectively. Under identical experimental conditions, the ED_50_ value of the positive control retigabine is 15.0 mg/kg [50].

### 4.3. Anti-Fungal, Anti-Parasitic and Anti-Viral Activities

In the recent years, both compounds and the crude extracts, such as volatile oil and ethyl acetate extracts, from CWW, CA, *C*. *komarovii* and other plants were investigated for their anti-fungal, parasitic or anti-viral activity, as shown below. 

Six compounds, namely, compounds **96**–**99**, **103** and **230** isolated from CWW roots, were evaluated against barley powdery mildew In vivo and compared with the anti-fungal activity of polyoxin B. The results suggested that compounds **98**, **99** and **103** exhibit potent In vivo anti-fungal activities and present disease-control values of >77% at a concentration of 63 μg/mL. The IC_50_ values (the concentration required for 50% inhibition) are 3.24, 12.90, and 28.35 μg/mL for compounds **99**, **103** and **98**, respectively [27].

Compound **20** was isolated from CA roots and was used to treat *Ichthyophthirius multifiliis*. This compound demonstrates 100% mortality rate of *I*. *multifiliis* in vitro after 5 h of exposure at 0.25 mg/L. The 5-hmedian effective concentration of compound to non-encysted tomonts is 0.083 mg/L [10].

Compounds **431**–**433** exhibit inhibitory activities against *Tobacco mosaic virus* (TMV). The results showed that alkaloids **432** and **433** exhibit anti-viral activities against TMV. The major active ingredient **432** exhibits 65% inhibition against the TMV at a concentration of 1.0 mg/mL. Alkaloid 433 shows 60% inhibition at 500 mg/mL, whereas compound **431** shows 15% inhibition at 500 mg/mL [92]. In comparison, 2,4-dioxo-hexahydro-1,3,5-triazine shows 50% inhibition at 500 mg/mL under the same conditions.

In addition, Yan et al. studied the anti-TMV activities of 42 compounds isolated from the roots of CA by using the conventional half-leaf method, enzyme-linked immunosorbent assay, and Western blot [36]. The results suggested that compounds **52**, **58**, **64**, **127** and **135** show significant anti-TMV activities with IC_50_ values of 20.5, 18.6, 22.0, 19.2 and 22.2 μg/mL, respectively. Moreover, the anti-TMV activities of these compounds are considerably more effective than that of the positive control, ningnanmycin (IC_50_ = 49.6 μg/mL).

The ethyl acetate extract of *C*. *paniculatum* exert an anti-viral effect against *Bovine viral diarrhea* (BVD) virus. The cytotoxic concentration (CC_50_ for the ethyl acetate extracts is 18.2 μg/mL. In the tissue culture infectious dose assay, the BVD virus decreased when treated with 18.2 μg/mL of the ethyl acetate extracts [107].

### 4.4. Anti-Inflammatory and Immunosuppressive Effects

Li et al. tested four C21 steroidal glycosides, namely, compounds **81**, **277**, **82** and **16**, for their immunological activities In vitro against concanavalin A (Con A)- and lipopolysaccharide (LPS)-induced proliferation of mice splenocytes [23]. The results showed that these compounds significantly inhibit the proliferation of Con A- and LPS-induced mice splenocytes in vitro in a dose-dependent manner.

Compound **120** has a significant inhibitory effect on TNF-*α* formation on the RAW 264.7 mouse macrophage-like cell line stimulated with LPS and N9 microglial cell line stimulated with LPS/IFN-*γ* (interferon-*γ*) [39].

Cho et al. investigated the anti-inflammatory effects and related molecular mechanisms of a crude polysaccharide (HMFO) which obtained from CWW in mice with dextran sulphate sodium (DSS)-induced colitis and in LPS-induced RAW 264.7 macrophages. It suggested that HMFO ameliorates the pathological characteristics of colitis and significantly reduces the production of proinflammatory cytokines in the serum [113]. Histological analysis indicated that HMFO improves the signs of histological damage. In addition, HMFO inhibits the protein expression levels of inducible nitric oxide synthase (iNOS) and cyclooxygenase-2 (COX-2) and phosphorylates the nuclear factor-kappa B (NF-*κ*B) p65 levels in the colon tissue of mice with DSS-induced colitis. In macrophages, HMFO inhibits several cytokines and enzymes involved in inflammation. HMFO also attenuates inflammation both in vitro and in vivo primarily by inhibiting NF-*κ*B activation.

Zhang et al. investigated the immunosuppressive activities of compounds **335–337** and **9** isolated from 80% ethanol extract of the CA root by using an In vitro model of Con A-induced proliferation of T lymphocytes from mice. As a result, these four compounds exhibit strong inhibition on Con A-stimulated cell proliferation, showing IC_50_ values of 3.3, 7.0, 6.7 and 10.9 μM [72]. In addition, compounds **341**–**346**, **348**–**350**, **352**–**354** and **356** were assessed for their immunological activities in vitro against Con A-induced proliferation of mice splenocytes [73]. The results revealed that compounds **341**, **342** and **354** at the concentration of 100 *μ*mol/L, compounds **343**, **352** and **354** at the concentration of 10 *μ*mol/L and compound **353** at the concentration of 1 *μ*mol/L exhibit weak activity against the proliferation of T lymphocyte In vitro.

Yu et al. found that compounds **358** and **359** inhibit nitric oxide production in C57bl/6j mouse peritoneal macrophages with 17.0% and 6.9% inhibition rates, respectively, at a concentration of 10 μM [74].

Fourteen steroidal glycosides were investigated by detecting the inhibitory effects of iNOS and COX-2 on RAW 246.7 murine macrophage cells stimulated by LPS [44]. The results revealed that compounds **158**, **162**, **156**, **157**, **122** and **146** can significantly inhibit iNOS expression, whereas compounds **162** and **148** can clearly inhibit COX-2 expression in RAW 246.7 cells stimulated by LPS compared with cells stimulated with LPS and not treated with other compounds.

The effects of compound **391** and extracts of CWW roots (CWE) on the expression of iNOS and proinflammatory cytokines in LPS-induced BV-2 microglial cells was investigated and the results suggested that CWE and compound **391** significantly decrease the LPS-induced NO production and the expression of iNOS in a concentration-dependent manner. Meanwhile, they did not show cytotoxic activity (CWE up to 500 μg/mL and compound **391** up to 80 μM). In addition, RT-PCR analysis and ELISA showed that compound **391** significantly attenuates the expression of TNF-*α*, interleukin-6, and interleukin -1*β* in LPS-stimulated BV-2 cells. Furthermore, compound **391** inhibits the phosphorylation of inhibitor kappa B-alpha and translocation of NF-*κ*B to the BV-2 cell nucleus. It indicates that CWE and compound **391** may exert effective anti-inflammatory activities via NF-*κ*B inactivation in stimulated microglial cells [110].

Choi et al. investigated the anti-atopic dermatitis (AD) effect and molecular mechanism of the aqueous extract of CA. Topical concentrations of CA at 1 and 100 mg/mL are applied to AD-like skin lesions induced by 2,4-dinitrochlorobenzene for 11 days. Scratching behavior occurrences were evaluated for 20 min. The results showed that topical application of CA attenuates the total serum IgE level [112].

### 4.5. Anti-Oxidizing Effect

Compound **419**, a steroidal alkaloid, was isolated from CWW roots, and its effects on lipid peroxidation and the activity of aldehyde oxidase (EC. 1.2.3.1) were investigated In vitro. The results showed that it suppresses the formation of lipid peroxides in rat liver tissues significantly and potently inhibits hepatic aldehydeoxidase activity in a dose-dependent manner, with a IC_50_ value of 0.8 μM (0.5 μg/mL) [94].

### 4.6. Hepatoprotective Function

Lee et al. investigated the hepatoprotective activity of compound **391** by using primary cultures of rat hepatocytes injured by CCl_4_. The results suggested that compound **391** (50 μM) significantly reduces (approximately 50%) the release into the culture medium of glutamic pyruvic transaminase and sorbitol dehydrogenase from the primary cultures of rat hepatocytes exposed to CCl_4_. Simultaneously, this compound ameliorates lipid peroxidation by up to 50%, as demonstrated by the reduction in malondialdehyde production [114]. In addition, Jang et al. found that CWE (100 and 200 mg/Kg) can decrease fat accumulation in the liver by suppressing COX-2, NF-κB and p38 mitogen-activated protein kinase [115].

### 4.7. Appetite Suppressant Effect

Compound **96** isolated from *C*. *auriculatum* roots can suppress appetite and reduce body weight in rats. Moreover, appetite suppressant isolated from *Hoodia gordonii* shows significant appetite suppressing effect, resulting in weight loss in rats [30].

### 4.8. Anti-Depressant Effect

Yang et al. assessed the anti-depressant activities of compounds **294**–**296**, **35** and **231** by using forced swimming, tail suspension and open field tests in despair mice models. The results suggested that these compounds show significant anti-depressant activities at the dosage of 50 mg/kg (i.g.). The most potential one is compound **295**, with potency close to that of the positive control fluoxetine (20 mg/kg) [67].

### 4.9. Vasodilating Activity

Compound **284** was isolated from the *C*. *stauntonii* roots, and its vasodilatation activity was investigated. The results indicated that this compound exerts a dose-dependent relaxation effect on aortic rings with endothelium contracted by phenylepherine, with IC_50_ value of 5.37 × 10^−6^ mol/L. The inhibitory effect of this compound on aortic rings with endothelium contracted by phenylepherine was exhibited by the relaxation effect at high concentration (10^−4^ mol/L), with a relaxation percentage 64.8% ± 26.9%. Meanwhile, compound **28** also relaxes the aorta rings contracted by KCl at high concentration (10^−4^ mol/L), with a relaxation percentage 53.4% ± 7.3% [40].

Moreover, Wang et al. [116] investigated the anti-angiogenic properties of compound **175** from *C*. *auriculatum*. The results revealed that it can significantly inhibit the proliferation of HUVEC human umbilical vein endothelial cell proliferation and block the HUVEC migration, invasion and capillary-like tube formation by disturbing the vascular endothelial growth factor (VEGF)-VEGFR2-protein kinase B (AKT)/focal adhesion kinase signal axis.

### 4.10. Others

In addition to the pharmacological activity of the above-mentioned reviewed *Cynanchum* plants, compound **394** from *C*. *bungei* exerts depigmenting activity [79]. Compounds **387** and **388** from *C*. *stauntonii* exhibit anti-cardiac congestion activity [77]. Compounds **392** and **394** have an anti-platelet effect [117]. Ten-week-old female rats were ovariectomized (OVX) and treated with the aqueous extract of CWW for 1 week. The administration of CWW (200 mg/kg/d for 7 days, per os) significantly improves skin temperature increase in OVX rats [119]. Moreover, the aqueous extract of CWW inhibits the development of benign prostatic hyperplasia (BPH) in a testosterone-induced BPH rat model [120]. In addition, compound **22** showed an airway smooth muscle relaxant effect [12].

## 5. Conclusions

*Cynanchum* L. is an important genus in the Asclepiadaceae family because numerous plants in this genus show several application prospects other than in the field of medicine. Moreover, *Cynanchum* plants present a long history as traditional folk medicine.

At present, more than 400 compounds have been isolated from genus *Cynanchum*. These compounds include steroids, flavonoids, acetophenones, triterpenoids, alkaloids, phytosterols, polysaccharides and other compounds. Among these compounds, C21 steroid is the characteristic ingredient. In China, several species have been used to treat chronic diseases in TCM for thousands of years, and the roots and stems of these species have been used as a component of TCM or in combination with other Chinese medicinal plants. 

Recently, increased attention has been focused on *C*. *taiwanianum*, *C*. *auriculatum*, *C*. *paniculatum*, CA, CWW, *C*. *otophyllum* and *C*. *stauntonii* because of their anti-tumor, neuroprotective, anti-fungal, parasitic and anti-viral, anti-depressant, anti-oxidant, anti-inflammatory and immunosuppressive effects. These plants also can suppress appetite, induce weight loss and expand blood vessels. 

Although a number of reports on the chemical components and pharmacological activities of these plants are available, studies on the chemical composition are still not systematic enough because they only focus on the chemical components of several species of this genus. However, research on the pharmacological activities are mostly based on in vitro activity screening, and pharmacodynamic studies in vivo represent only a few reports. Therefore, further investigations are required for systematic research of the chemical composition and in vivo pharmacological activities of *Cynanchum* sp. We believe that this work is of particular value by providing not only the fundamental insight into the medicinal value of plants in this genus; moreover, this work can provide reference for clinical medication, sustainable development and utilization of plants in this genus.

## Figures and Tables

**Table 1:** Traditional use of *Cynanchum* species in different regions of the world.

Name	Medicinal Parts	Traditional Uses	Distribution
*C. sibiricum* Willd.	Whole plant	Carbuncle swollen	Russia, China (Ovr Mongol, Gansu, Xinjiang)
*C. chinense* R. Br.	Whole plant	Wind-dispelling prescription	China (Liaoning, Hebei, Henan, Shandong, Shangxi, Ningxia, Gansu, Jiangsu, Zhejiang)
*C. auriculatum* Royle ex Wight	Roots	Stop coughing, cure neurasthenia, gastric and duodenal ulcers, nephritis, and so on.	India, China (Shandong, Hebei, Henan, Shanxi, Gansu, Tibet, Anhui, Jiangsu, Zhejiang, Fujian, Taiwan, Jiangxi, Hunan, Hubei, Guangxi, Guangdong, Guizhou, Sichuang, Yunnan)
*C. officinale* (Hemsl.) Tsiang et Zhang	Roots	Treatment of tonic analgesia, epilepsy, rabies and snake bites.	China (Shanxi, Anhui, Jiangxi, Hunan, Hubei, Guangxi, Guizhou, Sichuan, Yunnan)
*C. bungei* Decne.	Roots	For physically weak and insomnia, forgetful dreams, skin itching.	North Korea, China (Liaoning, OvrMongol, Hubei, Hunan, Shandong, Shanxi, Gansu).
*C. otophyllum* Schneid.	Roots	For rheumatoid bone pain, rubella itching, epilepsy, rabies bites, snake bites.	China (Hunan, Guangxi, Guizhou, Yunnan, Sichuan, Tibet)
*C. corymbosum* Wight	Whole plant	Treatment of neurasthenia, chronic nephritis, orchitis, urinary amenorrhea, tuberculosis, hepatitis and so on.	India, Burma, Laos, Vietnam, Kampuchea, Malaysia; China (Fujian, Guangxi, Guangdong, Sichuan, Yunnan)
*C. wilfordii* (Maxim.) Hemsl.	Roots	Injury, dysentery, infantile malnutrition, stomach pain, leucorrhea, sore ringworm.	China (Liaoning, Henan, Shandong, Shanxi, Shaanxi, Gansu, Xinjiang, Jiangsu, Anhui, Sichuan, Hunan, Hubei), North Korea, Japan.
*C. amplexicaule* (Sieb. et Zucc.) Hemsl. var. castaneum Makino	Whole plant	Swelling and poisoning, governance bruises, rheumatism.	North Korea, Japan, China (Heilongjiang, Liaoning)
*C. forrestii* Schltr. var. forrestii	Roots	Reduce pain, accelerate the healing.	Tibet, Gansu, Sichuan, Guizhou and Yunnan
*C. stauntonii* (Decne.) Schltr. ex Levl.	Whole plant	Treatment of lung disease, infantile malnutrition plot, cold cough and chronic bronchitis and so on.	Gansu, Anhui, Jiangsu, Zhejiang, Hunan, Jiangxi, Fujian, Guangdong, Guangxi and Guizhou.
*C. vincetoxicum* (L.) Pers.	Roots, seeds	Root: antiemetic; seed extract: treat cardiac failure.	China (Sichuan, Yunnan, Jiangsu and Taiwan), India and central and Western Europe
*C. inamoenum* (Maxim.) Loes.	Roots	Postpartum depression, pregnancy enuresis, scabies and lymphadenitis.	China (Liaoning, Hebei, Shandong, Shanxi, Anhui, Zhejiang, Hubei, Hunan, Shaanxi, Gansu, Guizhou, Sichuan, Tibet), North Korea and Japan.
*C. atratum* Bunge	Roots, stems	Clearing heat antitoxicant, insufficiency of vital energy and blood, fever.	China (Heilongjiang, Jilin, Shandong, Hebei, Henan, Shanxi, Shanxi, Sichuan, Guizhou, Yunnan, Guangxi, Liaoning, Guangdong, Hunan, Hubei, Fujian, Jiangxi, Jiangsu), North Korea and Japan
*C. glaucesces* (Decne.) Hand.-Mazz.	Roots, stems	Relieving dyspnea, antitussive and antiasthmatic.	Jiangsu, Zhejiang, Fujian, Jiangxi, Hunan, Guangdong, Guangxi and Sichuan
*C. paniculatum* (Bunge) Kitagawa	Roots, stems	Rheumatism, stomach pain, toothache, low back pain, flutters injury, urticaria, and eczema.	China (Liaoning, Ovr Mongol, Hebei, Henan, Shanxi, Gansu, Sichuan, Guizhou, Yunnan, Shandong, Anhui, Jiangsu, Zhejiang, Jiangxi, Shanxi, Hubei, Hunan, Guangdong and Guangxi), North Korea and Japan.
*C.versicolor* Bunge	Roots and stems	Reducing fever and causing diuresis, cure tuberculosis, edema, pain and so on.	China (Jilin, Liaoning, Hebei, Henan, Sichuan, Shandong, Jiangsu and Zhejiang)
*C. chekiangense* M. Cheng ex Tsiang et P. T. Li	Roots	Treatment of bruises, smashed topical, and scabies.	China (Zhejiang, Henan, Hunan and Guangdong)
*C.mooreanum* Hemsl.	Whole plant	Wash sores scabies.	China (Henan, Hubei, Hunan, Anhui, Jiangsu, Zhejiang, Jiangxi, Fujian and Guangdong)

Note: The above information was cited from the Chinese herbal and Chinese flora. References in this table was cited from the website: http://frps.eflora.cn/ and http://tool.zyy123.com/bencao/index.php.

**Table 2:** Popular traditional prescription composition of *Cynanchum* species.

Name	Compositions	Effect/Traditional Use	Ref.
Baiwei san	*Cynanchum atratum* Bunge, *Zingiber officinale* Rosc., *Trichosanthes kirilowii Maxim.*, Glycyrrhiza uralensis Fisch., Mirabilite.	Antidepressant	‘Qian jin yi fang’, vol. 18
Baiwei yuan	*Cynanchum atratum* Bunge, *Rehmannia glutinosa* (Gaetn.) Libosch. ex Fisch. et Mey., *Cinnamomum cassia* Presl, *Rubia yunnanensis* Diels, *Taxillus sutchuenensis* (Lecomte) Danser, *Dendrobium nobile* Lindl., *Achyranthes bidentata* Blume, *Ligusticum chuanxiong* Hort., *Saposhnikovia divaricata* (Trucz.) Schischk., *Panax ginseng* C. A. Mey., *Aristolochia fangchi* Y. C. Wu ex L. D. Chow et S. M. Hwang, *Cornus officinalis* Sieb. et Zucc., *Angelica sinensis* (Oliv.) Diels, *Schisandra chinensis* (Turcz.) Baill.	Infertility, abortion	‘Song·tai ping hui min he ji jv fang’
Baiwei tang	*Cynanchum atratum* Bunge, *Panax ginseng* C. A. Mey., *Angelica sinensis* (Oliv.) Diels, *Glycyrrhiza uralensis* Fisch.	Depressed dizziness, and occurrence of temporary fainting.	‘Pu ji ben shi fang’, vol. 7
Baiwei wan	*Cynanchum atratum* Bunge, *Panax ginseng* C. A. Mey., *Aconitum carmichaelii* Debx., *Rehmannia glutinosa* (Gaetn.) Libosch. ex Fisch. et Mey., *Cinnamomum cassia* Presl, *Cynanchum otophyllum* Schneid., *Evodia rutaecarpa* (Juss.) Benth., *Angelica sinensis* (Oliv.) Diels, *Areca catechu* L.	Irregular menstruation, infertility	‘Yi lve liu shu’, vol. 27
Baiwei gao	*Cynanchum atratum* Bunge, *Ampelopsis japonica* (Thunb.) Makino, *Bletilla striata* (Thunb. ex A. Murray) Rchb. f., *Typhonium giganteum* Engl., *Angelica dahurica* (Fisch. ex Hoffm.) Benth. et Hook. f. ex Franch. et Sav., *Paeonia lactiflora* Pall., frankincense, *Fraxinus chinensis* Roxb.	Evil sore	‘Shen hui’, vol. 63
Baiwei shiwei wan	*Cynanchum atratum* Bunge, *Anemarrhena asphodeloides* Bunge, Cortex Lycii, *Rehmannia glutinosa* (Gaetn.) Libosch. ex Fisch. et Mey., *Ophiopogon japonicus* (L.f.) Ker-Gawl., *Glycyrrhiza uralensis Fisch*, *Dichroa febrifuga* Lour., *Polygonatum odoratum* (Mill.) Druce, *Panax ginseng* C. A. Mey.	Frail, afraid of cold, heat	‘Wai tai’, vol. 3
Baiwei wan jiawei	*Saposhnikovia divaricata* (Trucz.) Schischk., *Notopterygium incisum* Ting ex H. T. Chang, *Cynanchum atratum* Bunge, *Tribulus terrester* L., pomegranate bark, *Taraxacum mongolicum* Hand.-Mazz., *Lonicera japonica* Thunb.	Breeze heat, Nasal obstruction, headache, fever	‘Shen shi yao han’
Buyi baiwei wan	*Cynanchum atratum* Bunge, *Dolomiaea souliei* (Franch.) Shih, *Angelica sinensis* (Oliv.) Diels, *Cinnamomum cassia* Presl, *Lycopuslucidus* Tur-Cz. var. hirtus Regel, *Achyranthes bidentata* Blume, *Rehmannia glutinosa* (Gaetn.) Libosch. ex Fisch. et Mey., *Paeonia suffruticosa* Andr., Panax ginseng C. A. Mey., *Ligusticum chuanxiong* Hort., *Atractylodes macrocephala* Koidz., *Citrus aurantium* L., *Asarum sieboldii* Miq., *Aconitum carmichaelii* Debx., *Astragalus membranaceus* (Fisch.) Bunge, *Dipsacus asperoides* C. Y. Cheng et T. M. Ai, *Evodia rutaecarpa* (Juss.) Benth., *Magnolia officinalis* Rehd. et Wils.	Postpartum weakness, pale complexion, diet reduced, increasingly thin.	‘Pu ji fang’, vol. 350
Jiawei baiwei wan	*Cynanchum atratum* Bunge, *Paeonia lactiflora* Pall., *Adenophora stricta* Miq., *Angelica sinensis* (Oliv.) Diels, *Ligusticum chuanxiong* Hort., *Glycyrrhiza uralensis* Fisch, *Astragalus membranaceus* (Fisch.) Bunge.	Too much blood loss, fainting	‘Wei sheng hong bao’, vol. 5
Huachong dingdan wan	*Rehmannia glutinosa* (Gaetn.) Libosch. ex Fisch. et Mey., *Cynanchum glaucescens* (Decne.) Hand.-Mazz.	Stomach pain	‘Bian zheng lu’, vol. 2
Xuanchaung weicha san	*Cynanchum atratum* Bunge, *Angelica dahurica* (Fisch. ex Hoffm.) Benth. et Hook. f. ex Franch. et Sav., *Daucus carota* L., *Stemona japonica* (Bl.) Miq., *Zanthoxylum bungeanum* Maxim., *Rehmannia glutinosa* (Gaetn.) Libosch. ex Fisch. et Mey.	Insecticide, detoxification	‘Yi liao bao jian cha tang pu’
Jiawei baiwei tang	*Cynanchum atratum* Bunge, *Semen Trichosanthis*, *Citrus maxima* (Burm.) Merr., *Fritillariae Thunbergii*, *Artemisia carvifolia*, *Dendrocalamopsis beecheyana* (Munro) Keng var. pubescens (P. F. Li) Keng f.	Pneumonia, cough	‘Ma pei zhi yi an’
Baiwei renshen wan	*Cynanchum atratum* Bunge, *Panax ginseng* C. A. Mey., *Rubia yunnanensis* Diels, *Achyranthes bidentata* Blume, *Asarum sieboldii* Miq., *Magnolia officinalis* Rehd. et Wils., *Pinellia ternata* (Thunb.) Breit., *Adenophora stricta* Miq., *Zingiber officinale* Rosc., *Gentiana macrophylla* Pall., *Zanthoxylum bungeanum* Maxim., *Angelica sinensis* (Oliv.) Diels, *Aconitum carmichaelii* Debx., *Saposhnikovia divaricata* (Trucz.) Schischk., *Aster tataricus* L. f.	Irregular menstruation, infertility	‘Qian jin yi fang’, vol. 2
Guizhi huangqi baiwei kuandonghua san	*Cinnamomum cassia* Presl, *Astragalus membranaceus* (Fisch.) Bunge, *Cynanchum atratum* Bunge, *Tussilago farfara* L., *Paeonia lactiflora* Pall., *Anemarrhena asphodeloides* Bunge.	Lung malaria	‘Jie nue lun shu’
Wumei baiwei xixin wan	*Dichroa febrifuga* Lour., *Cynanchum atratum* Bunge, *Clematis apiifolia* DC., *Anemarrhena asphodeloides* Bunge, *Sophora flavescens* Alt., *Dichroa febrifuga* Lour., *Glycyrrhiza uralensis* Fisch, *Asarum sieboldii* Miq.	Liver malaria	‘Jie nue lun shu’
Baiqian san	*Cynanchum glaucescens* (Decne.) Hand.-Mazz., *Glycyrrhiza uralensis* Fisch, *Panax ginseng* C. A. Mey., *Rehmannia glutinosa* (Gaetn.) Libosch. ex Fisch. et Mey., *Cannabis sativa* L., *Cinnamomum cassia* Presl, Wolfiporia cocos, *Astragalus membranaceus* (Fisch.) Bunge, donkey-hide gelatin, *Ophiopogon japonicus* (Linn. f.) Ker-Gawl.	Pulmonary fibrosis, cough and phlegm	‘Sheng hui’, vol. 31
Baiqian tang	*Cynanchum glaucescens* (Decne.) Hand.-Mazz., *Aster tataricus* L. f., *Pinellia ternata* (Thunb.) Breit., *Euphorbia pekinensis* Rupr.	Cough, body swollen, chest tightness, throat hoarse	‘Bei ji qian jin yao fang’, vol. 18
Baiqian yin	*Cynanchum glaucescens* (Decne.) Hand.-Mazz., *Platycodon grandiflorus* (Jacq.) A. DC., *Smilax china* L., *Amygdalus Communis Vas*, *Glycyrrhiza uralensis* Fisch.	Weak, cough, vomit blood	‘Sheng ji zong lu’, vol. 90
Shenyan baiqian tang	*Cynanchum glaucescens* (Decne.) Hand.-Mazz., *Pinellia ternata* (Thunb.) Breit., *Aster tataricus* L. f., *Ephedra sinica* Stapf, *Magnolia officinalis* Rehd. etWils., *Panax ginseng* C. A. Mey., *Glycyrrhiza uralensis* Fisch.	Cough, wheezing, nausea, vomiting, belching, hiccups	‘Sheng ji zong lu’, vol. 67
Xuchangqing san	*Cynanchum paniculatum* (Bunge) Kitagawa, *Sophora flavescens* Alt., *Aconitum carmichaelii* Debx.*, Evodia rutaecarpa* (Juss.) Benth., *Camptotheca acuminata* Decne., *Asarum sieboldii* Miq., *Acorus calamus* L., *Pinellia ternata* (Thunb.) Breit.	Scabies disease	‘Sheng ji zong lu’, vol. 137
Xuchangqing tang	*Cynanchum paniculatum* (Bunge) Kitagawa, *Perotis indica* (L.) Kuntze, *Akebia quinata* (Houtt.) Decne., *Malva crispa* Linn., *Areca catechu* L., *Dianthus superbus* L.	weakness of the spleen and the stomach	‘Ben cao gang mu’, vol. 13
Anwei jian	*Taraxacum mongolicum* Hand.-Mazz., *Cynanchum otophyllum* Schneid., *Glycyrrhiza uralensis* Fisch, *Carthamus tinctorius* L., *Cynanchum paniculatum* (Bunge) Kitagawa.	Stomach pain, blood circulation	‘Yuan zheng gang fang’
Huainan wan	*Plantago asiatica* L., *Prunus salicina* Lindl., *Adiantum capillus-veneris* L., *Cynanchum paniculatum* (Bunge) Kitagawa.	Tuberculosis, upset, headache and vomiting	‘Pu ji fang’, vol. 237

References in this table was cited from the website: http://www.wiki8.com.

**Table 3:** Compounds isolated from *Cynanchum* species.

No.	Compound Name	Species	Parts	Ref.
C21 steroids				
**1**	Cynanversicoside A	*C. versicolor*	Roots	[6]
**2**	Cynanversicoside B	*C. versicolor*	Roots	[6]
**3**	Cynanversicoside C	*C. versicolor*	Root/rhizome	[7]
**4**	Cynanversicoside D	*C. versicolor*	Root/rhizome	[7]
**5**	Cynanversicoside F	*C. versicolor*	Root/rhizome	[7]
**6**	Glaucogenin B	*C. glaucescens*	Roots	[8]
**7**	12*β*-*O*-(4-hydroxybenzoyl)-8*β*,14*β*,17*β*-trihydroxypregn-2,5-diene-20-one	*C. wilfordii*	Roots	[9]
**8**	12*β*-*O*-benzoyl-8*β*,14*β*,17*β*-trihydroxypregn-2,5-diene-20-one	*C. wilfordii*	Roots	[9]
**9**	Glaucoside A	*C. glaucescens*	Roots	[8]
**10**	Glaucoside B	*C. glaucescens*	Roots	[8]
**11**	Glaucoside C	*C. glaucescens*	Roots	[10]
**12**	Glaucoside D	*C. glaucescens*	Roots	[8]
**13**	Glaucoside E	*C. glaucescens*	Roots	[8]
**14**	Glaucoside F	*C. glaucescens*	Roots	[8]
**15**	Glaucoside G	*C. glaucescens*	Roots	[8]
**16**	Glaucoside H	*C. glaucescens*	Roots	[8]
**17**	Glaucoside I	*C. glaucescens*	Roots	[8]
**18**	Glaucoside J	*C. glaucescens*	Roots	[8]
**19**	Cynatratoside F	*C. atratum*	Roots	[8]
**20**	Cynatratoside C	*C. atratum*	Roots	[10]
**21**	Cynatratoside A	*C. atratum*	Roots	[11]
**22**	Cynatratoside B	*C. atratum*	Roots	[12]
**23**	Atratoside A	*C. atratum*	Roots	[13]
**24**	Atratoside B	*C. atratum*	Roots	[13]
**25**	Atratoside C	*C. atratum*	Roots	[14]
**26**	Atratoside D	*C. atratum*	Roots	[8]
**27**	Otophylloside A	*C. forrestii* *C. otophyllum* *C. wallichii*	Roots	[15]
**28**	Otophylloside B	*C. forrestii* *C. otophyllum* *C. wallichii*	Roots	[15]
**29**	Otophylloside C	*C. otophyllum*	Roots	[16]
**30**	Otophylloside F	*C. otophyllum*	Roots	[16]
**31**	Otophylloside H	*C. otophyllum*	Roots	[17]
**32**	Otophylloside I	*C. otophyllum*	Roots	[17]
**33**	Otophylloside J	*C. otophyllum*	Roots	[17]
**34**	Otophylloside K	*C. otophyllum*	Roots	[17]
**35**	Otophylloside L	*C. otophyllum* *C. auriculatum*	Roots	[17]
**36**	Otophylloside M	*C. otophyllum*	Roots	[17]
**37**	Otophylloside N	*C. forrestii*	Roots	[15]
**38**	Otophylloside O	*C. forrestii*	Roots	[15]
**39**	Otophylloside P	*C. forrestii*	Roots	[15]
**40**	Otophylloside Q	*C. forrestii*	Roots	[15]
**41**	Otophylloside R	*C. forrestii*	Roots	[15]
**42**	Otophylloside S	*C. forrestii*	Roots	[15]
**43**	Otophylloside T	*C. otophyllum*	Roots	[16]
**44**	Otophylloside U	*C. otophyllum*	Roots	[18]
**45**	Otophylloside V	*C. otophyllum*	Roots	[18]
**46**	Otophylloside W	*C. otophyllum*	Roots	[18]
**47**	Sibiricoside D	*C. sibiricum*	Roots	[19]
**48**	Sibiricoside E	*C. sibiricum*	Roots	[19]
**49**	Sibirigenin	*C. sibiricum*	Roots	[20]
**50**	Penupogenin	*C. sibiricum*	Roots	[20]
**51**	Penupogenin3-*O*-*β*-d-glucopyranosyl-(1→4)-*β*-l-cymaropyranosyl-(1→4)-*β*-d-cymaropyranosyl-(1→4)-*α*-l-diginopyranosyl-(1→4)-*β*-d-cymaropyranoside	*C. bungei*	Stems	[21]
**52**	Cynanoside A	*C. atratum*	Roots	[22]
**53**	Cynanoside B	*C. atratum*	Roots	[22]
**54**	Cynanoside C	*C. atratum*	Roots	[22]
**55**	Cynanoside D	*C. atratum*	Roots	[22]
**56**	Cynanoside E	*C. atratum*	Roots	[22]
**57**	Cynanoside F	*C. atratum*	Roots	[22]
**58**	Cynanoside G	*C. atratum*	Roots	[22]
**59**	Cynanoside H	*C. atratum*	Roots	[22]
**60**	Cynanoside I	*C. atratum* *C. versicolor*	Roots	[22]
**61**	Cynanoside J	*C. atratum*	Roots	[22]
**62**	Cynanoside K	*C. atratum*	Roots	[13]
**63**	Cynanoside L	*C. atratum*	Roots	[13]
**64**	Cynanoside M	*C. atratum*	Roots	[13]
**65**	Cynanoside N	*C. atratum*	Roots	[13]
**66**	Cynanoside O	*C. atratum*	Roots	[13]
**67**	Cynanosides P_1_	*C. atratum*	Roots	[14]
**68**	Cynanosides P_2_	*C. atratum*	Roots	[14]
**69**	Cynanosides P_3_	*C. atratum*	Roots	[14]
**70**	Cynanosides P_4_	*C. atratum*	Roots	[14]
**71**	Cynanosides P_5_	*C. atratum*	Roots	[14]
**72**	Cynanosides Q_1_	*C. atratum*	Roots	[14]
**73**	Cynanosides Q_2_	*C. atratum*	Roots	[14]
**74**	Cynanosides Q_3_	*C. atratum*	Roots	[14]
**75**	Cynanosides R_1_	*C. atratum*	Roots	[14]
**76**	Cynanosides R_2_	*C. atratum*	Roots	[14]
**77**	Cynanosides R_3_	*C. atratum*	Roots	[14]
**78**	Cynanoside S	*C. atratum*	Roots	[14]
**79**	Sublanceoside E_3_	*C. atratum*	Roots	[14]
**80**	Chekiangensoside A	*C. chekiangense*	Roots	[23]
**81**	Chekiangensoside B	*C. chekiangense*	Roots	[23]
**82**	Chekiangensoside C	*C. chekiangense*	Roots	[14]
**83**	Chekiangensoside D	*C. chekiangense*	Roots	[24]
**84**	Chekiangensoside E	*C. chekiangense*	Roots	[24]
**85**	Cynatroside A	*C. atratum*	Roots	[25]
**86**	Cynatroside B	*C. atratum*	Roots	[14]
**87**	Cynatroside C	*C. atratum*	Roots	[25]
**88**	Wilfoside A	*C. wilfordii*	Roots	[26]
**89**	Wilfoside B	*C. wilfordii*	Roots	[26]
**90**	Wilfoside C	*C. wilfordii*	Roots	[26]
**91**	Wilfoside D	*C. wilfordii*	Roots	[26]
**92**	Wilfoside E	*C. wilfordii*	Roots	[26]
**93**	Wilfoside F	*C. wilfordii*	Roots	[26]
**94**	Wilfoside G	*C. wilfordii*	Roots	[26]
**95**	Wilfoside H	*C. wilfordii*	Roots	[26]
**96**	Wilfoside KIN	*C.wilfordii*	Roots	[26]
**97**	Wilfoside K_1_GG	*C. wilfordii*	Roots	[27]
**98**	Wilfoside C_1_GG	*C. wilfordii*	Roots	[27]
**99**	Wilfoside C_1_N	*C. taiwanianum*	Roots	[28]
**100**	Wilfoside C_2_N	*C. taiwanianum*	Roots	[28]
**101**	Wilfoside C_3_N	*C. auriculatum*	Roots	[29]
**102**	Wilfoside M_1_N	*C. auriculatum*	Roots	[30]
**103**	Wilfoside C_1_G	*C. auriculatum*	Roots	[30]
**104**	Wilfoside C_2_G	*C. otophyllum*	Roots	[31]
**105**	Amplexicoside A	*C. amplexicaule*	Roots	[32]
**106**	Amplexicoside B	*C. amplexicaule*	Roots	[32]
**107**	Amplexicoside C	*C. amplexicaule*	Roots	[32]
**108**	Amplexicoside D	*C. amplexicaule*	Roots	[32]
**109**	Amplexicoside E	*C. amplexicaule*	Roots	[32]
**110**	Amplexicoside F	*C. amplexicaule*	Roots	[32]
**111**	Amplexicoside G	*C. amplexicaule*	Roots	[32]
**112**	Tylophoside A	*C. amplexicaule*	Roots	[32]
**113**	Hancoside A	*C. amplexicaule* *C. komarovii*	Roots	[33]
**114**	Hancoside	*C. forrestii* *C. hunmkiunum*	Roots	[34]
**115**	Neocynapanogenin F 3-*O-β*-d-thevetoside	*C. paniculatum*	Roots	[35]
**116**	Neocynapanogenin F	*C. paniculatum*	Roots	[35]
**117**	Neocynapanogenin F 3-*O*-*β*-d-thevetopyranoside	*C. atratum.*	Roots	[36]
**118**	Glaucogenin C	*C. hunmkiunum* *C. atratum*	Roots	[37]
**119**	Glaucogenin C 3-*O*-*α*-l-cymaropyranosyl-(1→4)-*β*-d-digitoxopyranosyl-(1→4)-*β*-d-canaropyranoside	*C. stauntonii*	root	[38]
**120**	Glaucogenin C 3-*O*-*β*-d-cymaropyranosyl-(1→4)-α-l-diginopyranosyl-(1→4)-*β*-d-thevetopyranoside	*C. atratum*	Roots	[39]
**121**	Glaucogenin C 3-*O*-*β*-d-thevetopyranoside	*C. atratum*	Roots	[39]
**122**	Glaucogenin C mono-d-thevetoside	*C. stauntonii*	Roots	[40]
**123**	Glaucogenin C 3-*O*-*β*-d-oleandropyranoside	*C. atratum.*	Roots	[36]
**124**	Glaucogenin C 3-*O*-*α*-l-diginopyranosyl-(1→4)-*β*-d-thevetopyranoside	*C. atratum.*	Roots	[36]
**125**	Glaucogenin C 3-*O*-*α*-l-cymaropyranosyl-(1→4)-*β*-d-cymaropyranosyl-(1→4)-*β*-d-oleandropyranoside	*C. atratum.*	Roots	[36]
**126**	Glaucogenin C 3-*O*-*α*-l-cymaropyranosyl-(1→4)-*β*-d-digitoxopyranosy-(1→4)-*β*-d-oleandropyranoside	*C. atratum.*	Roots	[36]
**127**	Glaucogenin C 3-*O*-*β*-d-cymaropyranosyl-(1→4)-*α*-l-diginopyranosyl-(1→4)-*β*-d-cymaropyranoside	*C. atratum.*	Roots	[36]
**128**	Glaucogenin C 3-*O*-*α*-d-oleandropyranosyl-(1→4)-*β*-d-digitoxopyranosyl-(1→4)-*β*-d-oleandropyranoside	*C. atratum.*	Roots	[36]
**129**	Glaucogenin C 3-*O*-*β*-d-thevetoside	*C. paniculatum.*	Root/rhizome	[41]
**130**	Glaucogenin A	*C. atratum.*	Roots	[36]
**131**	Glaucogenin A 3-*O*-*β*-d-oleandropyranoside	*C. atratum.*	Roots	[36]
**132**	Glaucogenin A 3-*O*-*β*-d-digitoxopyranoside	*C. atratum.*	Roots	[36]
**133**	Glaucogenin A 3-*O*-*β*-d-digitoxopyranosyl-(1→4)-*β*-d cymaropyranoside	*C. atratum.*	Roots	[36]
**134**	Glaucogenin A 3-*O*-*β*-d-glucopyranosyl-(1→4)-*β*-d-oleandropyranoside	*C. atratum.*	Roots	[36]
**135**	Glaucogenin A 3-*O*-*β*-d-cymaropyranosyl-(1→4)-*α*-l-diginopyranosyl-(1→4)-*β*-d-cymaropyranoside	*C. atratum.*	Roots	[36]
**136**	Glaucogenin A 3-*O*-*α*-l-cymaropyranosyl-(1→4)-*β*-d-digitoxopyranosyl-(1→4)-*β*-d-cymaropyranoside	*C. atratum.*	Roots	[36]
**137**	Glaucogenin A 3-*O*-*α*-d-oleandropyranosyl-(1→4)-*β*-d-digitoxopyranosyl-(1→4)-*β*-d-oleandropyranoside	*C. atratum.*	Roots	[36]
**138**	Glaucogenin A 3-*O*-*α*-l-cymaropyranosyl-(1→4)-*β*-d-digitoxopyranosyl-(1→4)-*β*-d-digitoxopyranoside	*C. atratum.*	Roots	[36]
**139**	Glaucogenin A 3-*O*-*β*-d-oleandropyranosyl-(1→4)-*β*-d-digitoxopyranosyl-(1→4)-*β*-d-oleandropyranoside	*C. atratum.*	Roots	[36]
**140**	Glaucogenin A 3-*O*-*α*-l-cymaropyranosyl-(1→4)-*β*-d-cymaropyranosyl-(1→4)-*β*-d-oleandropyranoside	*C. atratum.*	Roots	[36]
**141**	Glaucogenin A 3-*O*-*α*-l-cymaropyranosyl-(1→4)-*β*-d-cymaropyranosyl-(1→4)-*β*-d-cymaropyranoside	*C. atratum.*	Roots	[36]
**142**	Glaucogenin A 3-*O*-*β*-d-glucopyranosyl-(1→4)-*β*-d-glucopyranosyl-(1→4)-*β*-d-oleandropyranoside	*C. atratum.*	Roots	[36]
**143**	Glaucogenin A 3-*O*-*α*-l-oleandropyranosyl-(1→4)-*β*-d-digitoxopyranosyl-(1→4)-*β*-d-oleandropyranoside	*C. atratum.*	Roots	[36]
**144**	Glaucogenin D	*C. paniculatum.*	Root /rhizome	[41]
**145**	Stauntoside A	*C. stauntoi*	Roots	[42]
**146**	Stauntoside B	*C. stauntoi*	Roots	[42]
**147**	Stauntoside C	*C. stauntonii*	Roots	[43]
**148**	Stauntoside D	*C. stauntonii*	Roots	[43]
**149**	Stauntoside E	*C. stauntonii*	Roots	[43]
**150**	Stauntoside F	*C. stauntonii*	Roots	[43]
**151**	Stauntoside G	*C. stauntonii*	Roots	[43]
**152**	Stauntoside H	*C. stauntonii*	Roots	[43]
**153**	Stauntoside I	*C. stauntonii*	Roots	[43]
**154**	Stauntoside J	*C. stauntonii*	Roots	[43]
**155**	Stauntoside K	*C. stauntonii*	Roots	[43]
**156**	Stauntoside L	*C. stauntonii*	Roots	[44]
**157**	Stauntoside M	*C. stauntonii*	Roots	[44]
**158**	Stauntoside O	*C. stauntonii*	Roots	[44]
**159**	Stauntoside P	*C. stauntonii*	Roots	[44]
**160**	Stauntoside Q	*C. stauntonii*	Roots	[44]
**161**	Stauntoside R	*C. stauntonii*	Roots	[44]
**162**	Stauntoside S	*C. stauntonii*	Roots	[44]
**163**	Stauntoside T	*C. stauntonii*	Roots	[44]
**164**	Stauntoside UA	*C. stauntonii* .	Roots	[45]
**165**	Stauntoside UA_1_	*C. stauntonii* .	Roots	[45]
**166**	Stauntoside UA_2_	*C. stauntonii* .	Roots	[45]
**167**	Kidjoranin	*C. wilfordii.* *C. auriculatum*	Roots	[9]
**168**	Kidjoranin-3-*O*-*β*-d-oleandropyranosyl-(1→4)-*β*-d-oleandropyranosyl-(1→4)-*β*-d-cymaropyranosyl-(1→4)-*β*-d-cymaropyranoside	*C. otophyllum*	Roots	[46]
**169**	Kidjoranin 3-*O*-*β*-d-digitoxopyranoside	*C. otophyllum*	Roots	[47]
**170**	20-*O*-(4-hydroxybenzoyl)-kidjoranin	*C. wilfordii*	Roots	[9]
**171**	20-*O*-vanilloyl-kidjoranin	*C. wilfordii*	Roots	[9]
**172**	20-*O*-salicyl-kidjoranin	*C. wilfordii*	Roots	[9]
**173**	20-*O*-(4-hydroxybenzoyl)-kidjoranin	*C. wilfordii.*	Roots	[9]
**174**	12*β*-*O*-(4-hydroxybenzoyl)-8*β*,14*β*,17*β*-trihydroxypregn-2,5-diene-20-one	*C. wilfordii.*	Roots	[9]
**175**	Caudatin	*C. auriculatum*	Roots	[29]
**176**	caudatin-2,6-dideoxy-3-*O*-methy-*β*-d-cymaropyranoside	*C. auriculatum*	Roots	[48]
**177**	3-*O*-methyl-caudatin	*C. wilfordii*	Roots	[9]
**178**	Caudatin 3-*O*-*β*-d-glucopyranosyl-(1→4)-*β*-d-oleandropyranosyl-(1→4)-*β*-d-cymaropyranosyl-(1→4)-*β*-d-cymaropyranoside	*C. forrestii*	Roots	[15]
**179**	Caudatin 3-*O*-*α*-l-cymaropyranosyl-(1→4)-*α*-d-oleandropyranosyl-(1→4)-*α*-l-cymaropyranosyl-(1→4)-*β*-d-glucopyranosyl-(1→4)-*α*-d-oleandropyranosyl-(1→4)-*β*-d-oleandropyranosyl-(1→4)-*β*-d-diginopyranoside	*C. otophyllum*	Rhizome	[49]
**180**	Caudatin 3-*O*-*β*-d-cymaropyranosyl-(1→4)-*α*-d-oleandropyranosyl-(1→4)-*α*-l-cymaropyranosyl-(1→4)-*β*-d-glucopyranosyl-(1→4)-*β*-d-oleandropyranosyl-(1→4)-*β*-d-cymaropyranosyl-(1→4)-*β*-d-diginopyranoside	*C. otophyllum*	Rhizome	[49]
**181**	Caudatin 3-*O*-*β*-d-cymaropyranosyl-(1→4)-*β*-d-cymaropyranoside	*C. otophyllum*	Roots	[16]
**182**	Caudatin 3-*O*-*β*-d-glucopyranosyl-(1→4)-*β*-d-oleandropyranosyl-(1→4)-*β*-d-cymaropyranosyl-(1→4)-*β*-d-cymaropyranoside	*C. otophyllum*	Roots	[16]
**183**	Caudatin 3-*O*-*β*-d-glucopyranosyl-(1→4)-*β*-d-cymaropyranosyl-(1→4)-*α*-l-diginopyranosyl-(1→4)-*β*-d-cymaropyranoside	*C. wilfordii.*	Roots	[50]
**184**	Caudatin 3-*O*-*β*-d-cymaropyranosyl-(1→4)-*β*-d-oleandropyranosyl-(1→4)-*β*-d-cymaropyranoside	*C. otophyllum*	Roots	[46]
**185**	Caudatin-3-*O*-*β*-d-oleandropyranosyl-(1→4)-*β*-d-thevetopyranosyl-(1→4)-*β*-d-cymaropyranoside	*C. otophyllum*	Roots	[46]
**186**	Caudatin-3-*O*-*β*-d-thevetopyranosyl-(1→4)-*β*-d-cymaropyranosyl-(1→4)-*β*-d-cymaropyranoside	*C. otophyllum*	Roots	[46]
**187**	Caudatin-3-*O*-*β*-d-thevetopyranosyl-(1→4)-*β*-d-cymaropyranosyl-(1→4)-*β*-d-digitoxopyranoside.	*C. otophyllum*	Roots	[46]
**188**	Caudatin-3-*O*-*β*-d-cymaropyranosyl-(1→4)-*β*-d-oleandropyranosyl-(1→4)-*β*-d-cymaropyranosyl-(1→4)-*β*-d-digitoxopyranoside	*C. otophyllum*	Roots	[46]
**189**	Caudatin-3-*O*-*α*-l-cymaropyranosyl-(1→4)-*α*-d-cymaropyranosyl-(1→4)-*α*-l-cymaropyranosyl-(1→4)-*β*-d-digitoxopyranoside	*C. otophyllum*	Roots	[46]
**190**	Caudatin 3-*O*-*β*-d-oleandropyranosyl-(1→4)-*β*-d-thevetopyranosyl-(1→4)-*β*-d-cymaropyranosyl-(1→4)-*β*-d-cymaropyranoside	*C. otophyllum*	Roots	[46]
**191**	Caudatin 3-*O*-*α*-l-cymaropyranosyl-(1→4)-*β*-d-cymaropyranosyl-(1→4)-*α*-l-cymaropyranosyl-(1→4)-*β*-d-cymaropyranosyl-(1→4)-*β*-d-digitoxopyranoside.	*C. otophyllum*	Roots	[46]
**192**	Caudatin 3-*O*-*α*-l-cymaropyranosyl-(1→4)-*β*-d-cymaropyranosyl-(1→4)-*β*-d-cymaropyranosyl-(1→4)-*α*-l-cymaropyranosyl-(1→4)-*β*-d-oleandropyranosyl-(1→4)-*β*-d-cymaropyranoside	*C. otophyllum*	Roots	[46]
**193**	Caudatin 3-*O*-*β*-d-cymaropyranosyl-(1→4)-*β*-d-oleandropyranosyl-(1→4)-*β*-d-cymaropyranosyl-(1→4)-*β*-d-cymaropyranoside	*C. otophyllum*	Roots	[46]
**194**	Caudatin 3-*O*-*β*-d-oleandropyranosyl-(1→4)-*β*-d-digitoxopyranosyl-(1→4)-*β*-d-cymaropyranoside	*C. otophyllum*	Roots	[46]
**195**	Caudatin 3-*O*-*β*-d-oleandropyranosyl-(1→4)-*β*-d-cymaropyranosyl-(1→4)-*β*-d-digitoxopyranoside	*C. otophyllum*	Roots	[46]
**196**	Caudatin 3-*β*-d-digitoxopyranoside	*C. otophyllum*	Roots	[47]
**197**	Caudatin 3-*O*-*α*-l-diginopyranosyl-(1→4)-*β*-d-cymaropyranoside	*C. otophyllum*	Roots	[47]
**198**	Caudatin 3-*O*-*β*-d-glucopyranosyl-(1→4)-*β*-d-digitoxopyranosyl-(1→4)-*β*-d-diginopyranosyl-(1→4)-*α*-d-oleandropyranoside	*C. otophyllum*	Rhizome	[51]
**199**	Caudatin3-*O*-*β*-d-oleandropyranosyl-(1→4)-*α*-d-oleandropyranosyl-(1→4)-*α*-d-oleandropyranoside	*C. otophyllum*	Rhizome	[51]
**200**	Caudatin3-*O*-*β*-d-glucopyranosyl-(1→4)-*α*-d-oleandropyranosyl-(1→4)-*β*-d-diginopyranosyl-(1→4)-*α*-d-oleandropyranoside	*C. otophyllum*	Rhizome	[51]
**201**	Qingyangshengenin	*C. wilfordii.*	Roots	[9]
**202**	Qingyangshengenin 3-*O*-*β*-d-cymaropyranosyl-(1→4)-*β*-d-digitoxopyranoside	*C. otophyllum*	Roots	[16]
**203**	Qingyangshengenin 3-*O*-*β*-d-oleandropyranosyl-(1→4)-*β*-d-cymaropyranoside	*C. otophyllum*	Roots	[16]
**204**	Qingyangshengenin 3-*O*-*β*-d-oleandropyranosyl-(1→4)-*β*-d-cymaropyranosyl-(1→4)-*β*-d-digitoxopyranoside	*C. otophyllum*	Roots	[16]
**205**	Qingyangshengenin 3-*O*-*β*-d-cymaropyranosyl-(1→4)-*β*-d-thevetopyranosyl-(1→4)-*β*-d-cymaropyranosyl-(1→4)-*β*-d-digitoxopyranoside	*C. otophyllum*	Roots	[46]
**206**	Qingyangshengenin 3-*O*-*α*-l-cymaropyranosyl-(1→4)-*β*-d-oleandropyranosyl-(1→4)-*β*-d-cymaropyranosyl-(1→4)-*β*-d-cymaropyranoside	*C. otophyllum*	Roots	[46]
**207**	Qingyangshengenin 3-*O*-*α*-l-cymaropyranosyl-(1→4)-*β*-d-cymaropyranosyl-(1→4)-*β*-d-cymaropyranosyl-(1→4)-*α*-l-cymaropyranosyl-(1→4)-*β*-d-oleandropyranosyl-(1→4)-*β*-d-cymaropyranoside	*C. otophyllum*	Roots	[46]
**208**	Qinyangshengenin-3-*O*-*β*-d-oleandropyranosyl-(1→4)-*β*-d-oleandropyranosyl-(1→4)-*β*-d-cymaropyranosyl-(1→4)-*β*-d-cymaropyranoside	*C. otophyllum*	Roots	[52]
**209**	Qinyangshengenin-3-*O*-*α*-l-cymaropyranosyl-(1→4)-*β*-d-oleandropyranosyl-(1→4)-*β*-d-cymaropyranosyl-(1→4)-*β*-d-digitoxopyranoside	*C. wallichii*	Roots	[53]
**210**	Deacymetaplexigenin	*C. wilfordii*	Roots	[9]
**211**	12-*O*-vanilloyl-deacymetaplexigenin	*C. wilfordii.*	Roots	[9]
**212**	12-*O*-benzoyldeacymetaplexigenin	*C. wilfordii.*	Roots	[9]
**213**	17*β*-*O*-cinnamoyl-3*β*,8*β*,14*β*-trihydroxypregn-12,20-ether	*C. wilfordii.*	Roots	[9]
**214**	Gagamine 3-*O*-*β*-d-oleandropyranosyl-(1→4)-*β*-d-cymaropyranosyl-(1→4)-*β*-d-cymaropyranoside	*C. otophyllum*	Roots	[46]
**215**	Gagaminin 3-*O*-*β*-d-cymaropyranosyl-(1→4)-*β*-d-oleandropyranosyl-(1→4)-*β*-d-cymaropyranosyl-(1→4)-*β*-d-cymaropyranoside	*C. wilfordii*	Roots	[54]
**216**	Gagaminin 3-*O*-*β*-l-cymaropyranosyl-(1→4)-*β*-d-cymaropyranosyl-(1→4)-*α*-l-diginopyranosyl-(1→4)-*β*-d-digitoxopyranoside	*C. bungei*	Stems	[21]
**217**	Gagaminin 3-*O*-*β*-l-cymaropyranosyl-(1→4)-*β*-d-cymaropyranosyl-(1→4)-*α*-l-diginopyranosyl-(1→4)-*β*-d-cymaropyranoside	*C. bungei*	Stems	[21]
**218**	Gagaminine 3-*O*-*β*-d-oleandropyranosyl-(1→4)-*β*-d-oleandropyranosyl-(1→4)-*β*-d-cymaropyranoside	*C. saccatum*	Roots	[55]
**219**	Gagaminin 3-*O*-*α*-l-cymaropyranosyl-(1→4)-*β*-d-cymaropyranosyl-(1→4)-*α*-l-diginopyranosyl-(1→4)-*β*-d-digitoxopyranoside	*C. wilfordii.*	Roots	[50]
**220**	Gagaminin-3-*O*-*β*-d-oleandropyranosyl-(1→4)-*β*-d-cymaropyranosyl-(1→4)-*β*-d-digitoxopyranoside	*C. otophyllum*	Roots	[46]
**221**	12*β*-*O*-benzoyl-8*β*,14*β*,17*β*-trihydroxypregn-2,5-diene-20-one	*C. wilfordii.*	Roots	[9]
**222**	Rostratamin	*C. wilfordii.*	Roots	[9]
**223**	Rostratamine 3-*O*-*β*-d-oleandropyranosyl-(1→4)-*β*-d-cymaropyranosyl-(1→4)-*β*-d-cymaropyranoside	*C. otophyllum*	Roots	[16]
**224**	Sarcostin	*C. otophyllum*	Roots	[47]
**225**	12-*O*-nicotinoylsarcostin3-*O*-*β*-l-cymaropyranosyl-(1→4)-*β*-d-cymaropyranosyl-(1→4)-*α*-l-diginopyranosyl-(1→4)-*β*-d-cymaropyranoside	*C. bungei*	Stems	[21]
**226**	12-*O*-acetylsarcostin 3-*O*-*β*-lcymaropyranosyl-(1→4)-*β*-d-cymaropyranosyl-(1→4)-*β*-l-cymaropyranosyl-(1→4)-*β*-d-digitoxopyranosyl-(1→4)-*β*-d-digitoxopyranoside	*C. bungei*	Stems	[21]
**227**	12-*O*-acetylsarcostin3-*O*-*β*-l-cymaropyranosyl-(1→4)-*β*-d-digitoxopyranosyl-(1→4)-*β*-l-cymaropyranosyl-(1→4)-*β*-d-cymaropyranosyl-(1→4)-*α*-l-diginopyranosyl-(1→4)-*β*-d-cymaropyranoside	*C. bungei*	Stems	[21]
**228**	20-*O*-acetyl-12-*O*-cinnamoyl-3-*O*-(*β*-d-oleandropyranosyl-(1→4)-*β*-d-oleandropyranosyl-(1→4)-*β*-d-cymaropyranosyl)-8,14-secosarcostin-8,14-dione	*C. saccatum*	Roots	[56]
**229**	Deacylcynanchogenin	*C. wilfordii*	Roots	[9]
**230**	Cynauricuoside A	*C. wilfordii*	Roots	[27]
**231**	Cynauricuoside C	*C. auriculatum*	Root	[57]
**232**	Cynanside A	*C. aniculatum*	Roots	[58]
**233**	Cynanside B	*C. aniculatum*	Roots	[58]
**234**	Komaroside C	*C. forrestii*	Roots	[59]
**235**	Komaroside D	*C. komarovii*	Roots	[33]
**236**	Komaroside E	*C. komarovii*	Roots	[33]
**237**	Komaroside F	*C. komarovii*	Roots	[33]
**238**	Komaroside G	*C. komarovii*	Roots	[33]
**239**	Komaroside H	*C. komarovii*	Roots	[33]
**240**	Cynauricoside A	*C. wilfordii.*	Roots	[50]
**241**	Cynauricoside B	*C. auriculatum*	Roots	[30]
**242**	Cynauricoside C	*C. auriculatum*	Roots	[30]
**243**	Cynauricoside D	*C. auriculatum*	Roots	[30]
**244**	Cynauricoside E	*C. auriculatum*	Roots	[30]
**245**	Cynauricoside F	*C. auriculatum*	Roots	[30]
**246**	Cynauricoside G	*C. auriculatum*	Roots	[30]
**247**	Cynauricoside H	*C. auriculatum*	Roots	[30]
**248**	Cynauricoside I	*C. auriculatum*	Roots	[30]
**249**	Cynauricuside A	*C. auriculatum*	Roots	[30]
**250**	Cynaforroside B	*C. forrestii*	Roots	[59]
**251**	Cynaforroside C	*C. forrestii*	Roots	[59]
**252**	Cynaforroside D	*C. forrestii*	Roots	[59]
**253**	Cynaforroside E	*C. forrestii*	Roots	[59]
**254**	Cynaforroside F	*C. forrestii*	Roots	[59]
**255**	Cynaforroside G	*C. forrestii*	Roots	[59]
**256**	Cynaforroside H	*C. forrestii*	Roots	[59]
**257**	Cynaforroside I	*C. forrestii*	Roots	[59]
**258**	Cynaforroside J	*C. forrestii*	Roots	[59]
**259**	Cynaforroside K	*C. forrestii*	Roots	[60]
**260**	Cynaforroside L	*C. forrestii*	Roots	[60]
**261**	Cynaforroside M	*C. forrestii*	Roots	[60]
**262**	Cynaforroside N	*C. forrestii*	Roots	[60]
**263**	Cynaforroside O	*C. forrestii*	Roots	[60]
**264**	Cynaforroside P	*C. forrestii*	Roots	[60]
**265**	Cynaforroside Q	*C. forrestii*	Roots	[60]
**266**	Atratoglaucoside A	*C. atratum* *C. versicolor*	Roots	[39]
**267**	Atratoglaucoside B	*C. atratum*	Roots	[39]
**268**	Paniculatumoside A	*C. paniculatum* .	Roots	[61]
**269**	Paniculatumoside B	*C. paniculatum* .	Roots	[61]
**270**	Neohancoside C	*C. hunmkiunum*	Roots	[62]
**271**	Neohancoside D	*C. hunmkiunum*	Roots	[62]
**272**	Deoxyamplexicogenin A-3-*O*-yl-4-*O*-(4-*O*-*α*-l-cymaropyranosoyl-*β*-d-digitoxopyranosoyl)-*β*-d-canaropyranoside	*C. stauntonii*	Roots	[63]
**273**	2-deoxyamplexicogenin A	*C. stauntonii*	Roots	[64]
**274**	Amplexicogenin C-3-*O*-*β*-d-cymaropyranoside	*C. amplexicaule*	Roots	[65]
**275**	Cynascyroside A	*C. ascyrifolium*	Roots	[66]
**276**	Cynascyroside B	*C. ascyrifolium*	Roots	[66]
**277**	Cynascyroside C	*C. ascyrifolium* *C. chekiangense*	Roots	[66]
**278**	Cynascyroside D	*C. atratum*	Roots	[25]
**279**	Taiwanoside A	*C. taiwanianum*	Roots	[28]
**280**	Taiwanoside B	*C. taiwanianum*	Roots	[28]
**281**	Taiwanoside C	*C. taiwanianum*	Roots	[28]
**282**	Taiwanoside D	*C. taiwanianum*	Roots	[28]
**283**	Taiwanoside E	*C. taiwanianum*	Roots	[28]
**284**	Stauntonine	*C. stauntonii*	Roots	[40]
**285**	Anhydrohirundigenin	*C. stauntonii*	Roots	[40]
**286**	Anhydrohirundigenin monothevetoside	*C. stauntonii*	Roots	[40]
**287**	Auriculoside I	*C. auriculatum*	Roots	[29]
**288**	Auriculoside II	*C. auriculatum*	Roots	[29]
**289**	Auriculoside III	*C. auriculatum*	Roots	[29]
**290**	Auriculoside IV	*C. auriculatum*	Roots	[29]
**291**	Cynanauriculoside I	*C. auriculatum*	Roots	[29]
**292**	Cynanauriculoside II	*C. auriculatum*	Roots	[29]
**293**	Cynanauriculoside A	*C. wallichii*	Roots	[53]
**294**	Cynanauriculoside C	*C. auriculatum*	Roots	[67]
**295**	Cynanauriculoside D	*C. auriculatum*	Roots	[67]
**296**	Cynanauriculoside E	*C. auriculatum*	Roots	[67]
**297**	(3*β*,8*β*,9*α*,16*α*,17*α*)-14,16*β*:15,20*α*:18,20*β*-triepoxy-16*α*,17*α*-dihydroxy-14-oxo-13,14:14,15-disecopregna-5,13(18)-dien-3-yl *α*-cymaropyranosyl-(1→4)-*α*-digitoxopyranosyl-(1→4)-*α*-oleandropyranoside	*C. paniculatum*	Stems	[68]
**298**	(3*β*,8*β*,9*α*,16*α*,17*α*)-14,16*β*:15,20*α*:18,20*β*-triepoxy-16*β*:17*α*-dihydroxy-14-oxo-13,14:14,15-disecopregna-5,13(18)-dien-3-yl *α*-oleandropyranosyl-(1→4)-*α*-digitoxopyranosyl-(1→4)-*α*-oleandropyranoside	*C. paniculatum*	Stems	[68]
**299**	Cyanoauriculoside C	*C. auriculatum*	Roots	[69]
**300**	Cyanoauriculoside D	*C. auriculatum*	Roots	[69]
**301**	Cyanoauriculoside E	*C. auriculatum*	Roots	[69]
**302**	Cyanoauriculoside G	*C. wilfordii.*	Roots	[50]
**303**	Hirundoside A	*C. stauntonii*	Roots	[43]
**304**	Deacetylmetaplexigenin	*C. otophyllum*	Roots	[47]
**305**	Deacetylmetaplexigenin 3-*O*-*β*-d-oleandropyranosyl-(1→4)-*α*-d-oleandropyranosyl-(1→4)-*α*-d-oleandropyranoside	*C. otophyllum*	Rhizome	[70]
**306**	Deacetylmetaplexigenin 3-*O*-*α*-d-oleandropyranosyl-(1→4)-*β*-d-thevetopyranosyl-(1→4)-*α*-d-oleandropyranoside	*C. otophyllum*	Rhizome	[70]
**307**	Deacetylmetaplexigenin 3-*O*-*β*-d-cymaropyranosyl-(1→4)-*α*-d-oleandropyranoside	*C. otophyllum*	Rhizome	[70]
**308**	Cynsaccatol A	*C. saccatum*	Roots	[55]
**309**	Cynsaccatol B	*C. saccatum*	Roots	[55]
**310**	Cynsaccatol C	*C. saccatum*	Roots	[55]
**311**	Cynsaccatol D	*C. saccatum*	Roots	[55]
**312**	Cynsaccatol E	*C. saccatum*	Roots	[55]
**313**	Cynsaccatol F	*C. saccatum*	Roots	[55]
**314**	Cynsaccatol G	*C. saccatum*	Roots	[55]
**315**	Cynsaccatol H	*C. saccatum*	Roots	[55]
**316**	Cynotophylloside A	*C. otophyllum.*	Roots	[47]
**317**	Cynotophylloside B	*C. otophyllum.*	Roots	[47]
**318**	Cynotophylloside C	*C. otophyllum.*	Roots	[47]
**319**	Cynotophylloside D	*C. otophyllum.*	Roots	[47]
**320**	Cynotophylloside E	*C. otophyllum.*	Roots	[47]
**321**	Cynotophylloside F	*C. otophyllum.*	Roots	[47]
**322**	Cynotophylloside H	*C.otophyllum*	Roots/stems	[71]
**323**	Stephanoside H	*C. otophyllum*	Roots	[46]
**324**	Wallicoside	*C. otophyllum*	Roots	[18]
**325**	Wallicoside J	*C. otophyllum*	Roots	[46]
**326**	Cynawilfoside A	*C. wilfordii.*	Roots	[50]
**327**	Cynawilfoside B	*C. wilfordii.*	Roots	[50]
**328**	Cynawilfoside C	*C. wilfordii.*	Roots	[50]
**329**	Cynawilfoside D	*C. wilfordii.*	Roots	[50]
**330**	Cynawilfoside E	*C. wilfordii.*	Roots	[50]
**331**	Cynawilfoside F	*C. wilfordii.*	Roots	[50]
**332**	Cynawilfoside G	*C. wilfordii.*	Roots	[50]
**333**	Cynawilfoside H	*C. wilfordii.*	Roots	[50]
**334**	Cynawilfoside I	*C. wilfordii.*	Roots	[50]
**335**	Atratcynoside A	*C. atratum*	Roots	[72]
**336**	Atratcynoside B	*C. atratum*	Roots	[72]
**337**	Atratcynoside C	*C. atratum*	Roots	[72]
**338**	Atratcynoside D	*C. atratum*	Roots	[72]
**339**	Atratcynoside E	*C. atratum*	Roots	[72]
**340**	Atratcynoside F	*C. atratum*	Roots	[72]
**341**	Mooreanoside A	*C. mooreanum*	Roots	[73]
**342**	Mooreanoside B	*C. mooreanum*	Roots	[73]
**343**	Mooreanoside C	*C. mooreanum*	Roots	[73]
**344**	Mooreanoside D	*C. mooreanum*	Roots	[73]
**345**	Mooreanoside E	*C. mooreanum*	Roots	[73]
**346**	Mooreanoside F	*C. mooreanum*	Roots	[73]
**347**	Mooreanoside G	*C. mooreanum*	Roots	[73]
**348**	Mooreanoside H	*C. mooreanum*	Roots	[73]
**349**	Mooreanoside I	*C. mooreanum*	Roots	[73]
**350**	Mooreanoside J	*C. mooreanum*	Roots	[73]
**351**	Mooreanoside K	*C. mooreanum*	Roots	[73]
**352**	Mooreanoside L	*C. mooreanum*	Roots	[73]
**353**	Mooreanoside M	*C. mooreanum*	Roots	[73]
**354**	Mooreanoside N	*C. mooreanum*	Roots	[73]
**355**	Mooreanoside O	*C. mooreanum*	Roots	[73]
**356**	Mooreanoside P	*C. mooreanum*	Roots	[73]
**357**	Cynastauoside A	*C. stauntonii*	Roots	[74]
**358**	Cynastauoside B	*C. stauntonii*	Roots	[74]
**359**	Cynastauoside C	*C. stauntonii*	Roots	[74]
**360**	Saccatol A	*C. saccatum*	Roots	[56]
**361**	Saccatol B	*C. saccatum*	Roots	[56]
**362**	Saccatol C	*C. saccatum*	Roots	[56]
**363**	Cynanotoside A	*C. otophyllum*	Roots/stems	[71]
**364**	Cynanotoside B	*C. otophyllum*	Roots/stems	[71]
**365**	Cynanotoside C	*C. otophyllum*	Roots/stems	[71]
**366**	Cynanotoside D	*C. otophyllum*	Roots/stems	[71]
**367**	Cynanotoside E	*C. otophyllum*	Roots/stems	[71]
**368**	Mucronatoside C	*C. otophyllum*	Roots	[46]
**369**	Sinomarinoside B	*C. otophyllum*	Roots	[46]
**370**	Cynanotophylloside A	*C. otophyllum*	Roots	[31]
**371**	Cynanotophylloside B	*C. otophyllum*	Roots	[31]
**372**	Cynanotophylloside C	*C. otophyllum*	Roots	[31]
**373**	Cynanotophylloside D	*C. otophyllum*	Roots	[31]
**374**	Cynanauriculatoside A	*C. otophyllum*	Roots	[31]
**375**	3*β*,14*β*-dihydroxy-14*β*-pregn-5-en-20-one	*C. paniculatum.*	Root/rhizome	[41]
**376**	3-*O*-*β*-d-oleandropanyanoside	*C. paniculatum.*	Root/rhizome	[41]
**377**	Hancopregnane	*C. hunmkiunum*	Roots	[37]
**378**	Menarandroside A	*C. menarandrense*	Aerial parts	[75]
**379**	Menarandroside B	*C. menarandrense*	Aerial parts	[75]
**380**	Menarandroside C	*C. menarandrense*	Aerial parts	[75]
**381**	Menarandroside D	*C. menarandrense*	Aerial parts	[75]
**382**	Menarandroside E	*C. menarandrense*	Aerial parts	[75]
**383**	Carumbelloside I	*C. menarandrense*	Aerial parts	[75]
**384**	Carumbelloside II	*C. menarandrense*	Aerial parts	[75]
**385**	Pregnenolone-3-*O*-gentiobioside	*C. menarandrense*	Aerial parts	[75]
**386**	14-*O*-methyl-3-epi-hirundigenin	*C. stauntonii*	Roots	[76]
**387**	Stauntosaponin A	*C. stauntonii*	Roots	[77]
**388**	Stauntosaponin B	*C. stauntonii*	Roots	[77]
**Benzene and its derivatives**				
**389**	Cynantetrone	*C. taiwanianum*	Rhizome	[78]
**390**	CynantetroneA	*C. taiwanianum*	Rhizome	[78]
**391**	Cynandione A	*C. taiwanianum*	Rhizome	[78]
**392**	Cynandione B	*C. taiwanianum*	Rhizome	[78]
**393**	2,4-Dihydroxyacetophenone	*C. atratum*	Roots	[25]
**394**	2,5-Dihydroxyacetophenone	*C. bungei*	Roots	[79]
**395**	4-Hydroxyacetophenone	*C. atratum*	Roots	[25]
**396**	4-acetylphenol	*C. paniculatum*	Roots	[80]
**397**	2,5-dihydroxy-4-methoxyacetophenone	*C. paniculatum*	Roots	[80]
**398**	2,3-dihydroxy-4-methoxyacetophenone	*C. paniculatum*	Roots	[81]
**399**	Acetoveratrone	*C. paniculatum*	Roots	[80]
**400**	2,5-dimethoxyhydroquinone	*C. paniculatum*	Roots	[80]
**401**	Resacetophenone	*C. paniculatum*	Roots	[80]
**402**	M-acetylphenol	*C. paniculatum*	Roots	[80]
**403**	Vanillic acid	*C. paniculatum*	Roots	[80]
**404**	3,5-dimethoxyhydroquinone	*C. paniculatum*	Roots	[80]
**405**	Acetovanillone	*C. wilfordii*	Roots	[3]
**406**	p-hydroxyacetophenone	*C. wilfordii*	Roots	[3]
**407**	3-(*β*-d-ribofuranosyl)-2,3-dihydro-6H-1,3-oxazine-2,6-dione	*C. wilfordii*	Roots	[3]
**408**	Bungeiside A	*C. wilfordii*	Roots	[3]
**409**	Cynanoneside B	*C. wilfordii*	Roots	[3]
**410**	Cynanoneside A	*C. taiwanianum*	Roots	[82]
**411**	Baishouwubenzophenone	*C. auriculatum*	Roots	[83]
**412**	3,4-dihydroxyacetophenone	*C. atratum*	Roots	[39]
**413**	4′-hydroxy-3′-methoxyacetophenone	*C. wilfordii*	Roots	[84]
**414**	Paeonol	*C. auriculatum*	Roots	[58]
**415**	Isopaeonol	*C. auriculatum*	Roots	[58]
**416**	2-hydroxy-5-methoxyacetophenone	*C. auriculatum*	Roots	[58]
**417**	Caffeic acid	*C. taiwanianum*	Aerial parts	[85]
**418**	Syringic acid	*C. paniculatum*	Roots	[86]
**Alkaloids**				
**419**	Gagamine	*C. caudatum*	Roots	[87]
**420**	Antofine	*C. vincetoxicum*	Aerial parts	[88]
**421**	Tylophorine	*C. vincetoxicum*	Aerial parts	[88]
**422**	Vincetene	*C. vincetoxicum*	Aerial parts	[88,89]
**423**	(-)-10*β*,13a*α*-14*β*-hydroxyantofine *N*-oxide	*C. vincetoxicum*	Aerial parts	[90]
**424**	(-)-10*β*,13a*α*-secoantofine *N*-oxide	*C. vincetoxicum*	Aerial parts	[90]
**425**	(-)-(*R*)-13a*α*-6-*O*-desmethylantofine	*C. vincetoxicum*	Aerial parts	[91]
**426**	(-)-(*R*)-13a*α*-secoantofine	*C. vincetoxicum*	Aerial parts	[91]
**427**	(-)-(*R*)-13a*α*-6-*O*-desmethylsecoantofine	*C. vincetoxicum*	Aerial parts	[91]
**428**	(-)-10*β*-antofine *N*-oxide	*C. vincetoxicum*	Aerial parts	[90]
**429**	2,3-dimethoxy-6-(3-oxo-butyl)-7,9,10,11,11a,12-hexahydrobenzo[*f*]pyrrolo[*1*,*2-b*]isoquinoline	*C. komarovii*	Aerial parts	[92]
**430**	7-demethoxytylophorine	*C. komarovii*	Aerial parts	[92]
**431**	7-demethoxytylophorine *N*-oxide	*C. komarovii*	Aerial parts	[92]
**Flavones**				
**432**	7-*O*-*α*-l-rhamnopyranosyl-kaempferol-3-*O*-*β*-d-glucopyranoside	*C. chinese*	Aerial parts	[93]
**433**	7-*O*-*α*-l-rhamnopyranosyl-kaempferol-3-*O*-*α*-l-rhamnopyranoside	*C. chinese*	Aerial parts	[93]
**434**	Kaempferol	*C. taiwanianum*	Aerial parts	[85]
**435**	Astragalin	*C. taiwanianum*	Aerial parts	[85]
**436**	Afzelin	*C. taiwanianum*	Aerial parts	[85]
**437**	Trifolin	*C. taiwanianum*	Aerial parts	[85]
**438**	Quercetin	*C. taiwanianum*	Aerial parts	[85]
**439**	Isoquercitrin	*C. taiwanianum*	Aerial parts	[85]
**440**	Quercitrin	*C. taiwanianum*	Aerial parts	[85]
**441**	Hyperin	*C. taiwanianum*	Aerial parts	[85]
**Terpene**				
**442**	Neohancoside A	*C. hunmkiunum*	Roots	[34]
**443**	Neohancoside B	*C. hunmkiunum*	Roots	[62]
**444**	*β*-amyrin	*C. paniculatum*	Roots	[86]
**445**	*α*-amyrin	*C. paniculatum*	Roots	[86]
**446**	Lupeol	*C. paniculatum*	Roots	[86]
**447**	Taraxasterol	*C. paniculatum*	Roots	[86]
**448**	Ursolic acid	*C. paniculatum*	Roots	[86]
**449**	Oleanolic acid	*C. paniculatum*	Roots	[86]
**450**	Maslinic acid	*C. paniculatum*	Roots	[86]

**Table 4:** Summary of pharmacological activities of the extracts/compounds from different parts of *Cynanchum* species.

Cynanchum Species	Extract/Isolate	Plant Part	In Vitro/In Vivo	Dosage/Duration	Model/Effect	Ref.
Anti-cancer						
***C. taiwanianum***	Cynantetrone, cynandione B	Rhizome	In vitro		Compounds against T-24 cell lines with ED_50_ values of ca. 3.5 and 2.5 μg/mL, respectively, and cynandione B against PLC/PRF/5 cell lines (ED_50_ = 2.7 μg/mL).	[78]
***C.auriculatum***	Ethanol extract, Petroleumether, CHCl_3_, EtOAc and *n*-BuOH fraction	Root tubers	In vitro	1 μg/mL	The ethanol extract against K562, with the highest inhibition ratio of 24.06% at a concentration of 1 μg/mL.	[96]
			In vivo	100 mg/kg/Gavage 7 d	The ethanol extract and n-BuOH fraction showed significant antitumor activity by inhibiting the growth of sarcoma S180 in mice with an inhibition ratio of 42.22% and 41.50%.	
***C. auriculatum ***	Total glucosides		In vivo	225 mg/kg 10 d	Model: C57BL/6 mice bearing Lewis lung carcinoma. The inhibition rate of tumor weight was 38.68% the inhibition rate of lung metastasis was 63.64%.	[97]
***C.auriculatum ***	Caudatin, caudatin-2,6-dideoxy-3-*O*-methy-*β*-d-cymaropyranoside	Root tubers	In vitro	12 μM	Model: Human tumor cell line SMMC–7721. IC_50_ = 24.95 μM; IC_50_ = 13.49 μM	[48]
			In vivo	10, 20, 40 mg/Kg 9 d	Model: Transplantable H22 tumors in mice. The growth of transplantable H22tumors in mice was inhibited.	
***C.auriculatum***	Kidjoranin 3-*O*-*α*-diginopyranosyl-(1→4)-*β*-cymaropyranoside, kidjoranin 3-*O*-*β*-digitoxopyranoside, caudatin 3-*O*-*β*-cymaropyranoside	Roots	In vitro		Model: SMMC-7721 and HeLa cell lines. IC_50_ = 8.6 μM–58.5 μM.	[98]
***C. auriculatum***	Auriculoside A, auriculoside B	Roots	In vitro		Have significant cytoxicity against PC3, Hce-8693, Hela, and PAA cell lines.	[99]
***C. vincetoxicum***	Alkaloids	Overground	In vitro		These alkaloids inhibit growth of the hormone in dependent breast cancer cells MDA-MB 231.	[88]
***C. paniculatum***	Neocynapanogenin F, neocynapanogenin F 3-*O*-*β*-d-thevetoside	Roots	In vitro	100 μg/mL	These compounds exhibited significant cytotoxic activity on HL-60. The inhibitory rate (%, *n* = 6) was 74.18% and 97.87%, respectively.	[35]
***C. paniculatum***	Cynanside A, Cynanside B	Roots	In vitro		Model: SK-MEL-2 cells. IC_50_ values = 26.55 μM; IC_50_ values = 17.36 μM	[58]
***C. paniculatum***	Antofine	Roots	In vitro	Ellipticine: IC_50_ = 500 ± 25 ng/mL	Model: Human lung cancer cells A549. IC_50_ = 7.0 ± 0.2 ng/mL	[100]
				Ellipticine: IC_50_ = 340 ± 35 ng/mL	Model: Human colon cancer cells Col2. IC_50_ = 8.6 ± 0.3 ng/mL	
***C. wilfordii***	20-*O*-salicyl-kidjoranin	Roots	In vitro	Adriamycin	Model: Human leukemia cell lines HL-60, K562 and breast cancer cell lines MCF-7. The compound can against HL-60 (IC_50_ = 6.72 μM) and MCF-7 (IC_50_ = 2.89 μM).	[9]
	Qingyangshengenin				The compound can against K-562 (IC_50_ = 6.72 μM).	
	Rostratamin				The compound can against MCF-7 (IC_50_ = 2.49 μM).	
***C. wilfordii***	Gagaminin 3-*O*-*β*-d-cymaropyranosyl-(1→4)-*β*-d-oleandropyranosyl-(1→4)-*β*-d-cymaropyranosyl-(1→4)-*β*-d-cymaropyranoside	Roots	In vitro	1 μM	Model: KB-V1 and MCF7/ADR cells. The compounds completely reverse the multidrug-resistance of KB-V1 and MCF7/ADR cells to Adriamycin, vinblastine, and colchicine.	[54]
***C. atratum***	Glaucogenin C 3-*O*-*β*-d-cymaropyranosyl-(1→4)-*α*-l-diginopyranosyl-(1→4)-*β*-d-thevetopyranoside	Roots	In vitro	Dexamethasone: 10 μM, compound: 30 μM	Model: 212 cells, RAW 264.7 mouse macrophage-like cell, N9 microglial cell. ED_50_ value of against 212 cells was 0.96 μg/mL and significant inhibitory on TNF-*α *formation.	[39]
***C. vincetoxicum***	(-)-10*β*-antofine *N*-oxide , (-)-10*β*,13a*α*-14*β*-hydroxyantofine *N*-oxide	Aerial parts	In vitro		Model: drug-sensitive KB-3-1 cell line and the multi-drug-resistant KB-V1 cell line. IC_50_ = 100 nM	[90]
***C. vincetoxicum***	(-)-(*R*)-13a*α*-antofine, (-)-(*R*)-13a*α*-6-*O*-desmethylantofine	Leaves	In vitro		Model: KB-3-1 and the KB-V1 cell line. IC_50_ values of 7–17 nM	[91]
***C. saccatum***	Cynsaccatol E	Roots	In vitro	5-FU and cisplatin	Model: HepG2 cell lines IC_50_ = 49.18 ± 5.67μM.	[55]
	Gagaminine 3-*O*-*β*-d-oleandropyranosyl-(1→4)-*β*-d-oleandropyranosyl-(1→4)-*β*-d-cymaropyranoside				Model: HepG2 and Hela cell lines. IC_50_ = 68.05 ± 4.09 μM and IC_50_ = 94.88 ± 9.73 μM.	
	Cynsaccatol A				Model: U251 cell lines. IC_50_ = 35.66 ± 3.54 μM.	
	Cynsaccatol D				Model: U251 cell lines. IC_50_ = 31.98 ± 6.55 μM	
***C. saccatum***	Glaucogenin C-3-*O*-*β*-d-monothevetoside	Whole fresh plants	In vitro	Cisplatin: IC_50_ = 21.51 μM	The compound could induce HepG2 cell apoptosis via a mitochondrial pathway and IC_50_ value of 12.24 μM	[101]
***C. paniculatum***	Cynatratoside B	Roots	In vitro	5-Fluorouracil	Compound exhibited potent inhibitory activities against HL-60, HT-29, PC-3 and MCF-7 cell lines with IC_50_ values of 8.3, 7.5, 34.3 and 19.4 μM, respectively.	[102]
***C. atratum***	C_21_ steroids	Roots	In vitro	Cisplatin (25 μg/mL)	Model: HepG2, A549 cell lines. Compounds **1**–**4** displayed obvious cytotoxic activities against HepG2 cells with IC_50_ values ranging from 10.19 μM to 76.12 μM. Compounds **1**–**3** also exhibited cytotoxic effects in A549 cells with IC_50 _values of 30.87–95.39 μM.	[103]
**Neuroprotective effect**						
***C. wilfordii***	Cynandione A	Roots	In vitro	50 μM.	Model: Neurotoxicity induced by H_2_O_2_ in cultured cortical cells. The compound could reduce neurotoxicity induced by H_2_O_2._	[104]
***C. atratum***	Cynatroside A, cynatroside B, cynatroside C, cynascyroside D	Roots	In vitro	Velnacrine: IC_50_ = 0.4 μM.	These compounds could inhibit acetylcholinesterase activity. IC_50_ = 6.4 μM, IC_50_ = 3.6 μM, IC_50_ = 52.3 μM, IC_50_ = 52.9 μM, respectively.	[25]
***C. paniculatum***	2,3-dihydroxy-4-methoxyacetophenone	Roots	In vitro	Trolox (10 μM).	Model: Glutamate-induced neurotoxicity in HT22 cells. Relatively effective protection of 47.55% (at 10 μM).	[81]
***C. atratum***	Cynatroside B	Roots	In vivo	Donepezil: 0.032–3.2 mg/Kg body weight i.p.	The results showed that compound has both anti-AchE and anti-amnesic activities.	[105]
***C. otophyllum***	Cynanotoside A, cynanotoside B, cynotophylloside H	Roots and stems	In vitro		Three oxidative stress models induced by glutamate, H_2_O_2_, and homocysteic acid (HCA), respectively, in a hippocampal neuronal cell line HT22. Compounds showed significant dose-dependent protection to HCA-induced cell death ranging from 1 to 30 μM.	[71]
***C. otophyllum***	Otophylloside F, otophylloside B	Roots	In vivo	phenytoin sodium showed a therapeutic efficacy of 66% at 300 μM	Model: Antiseizure-like locomotor activity in the zebrafish bioassay model. The otophylloside F at a 300 μM concentration showed a therapeutic efficacy of 55%. The otophylloside B at 100 and 200 μM concentrations showed therapeutic efficacies of 77% and 90%, respectively.	[16]
***C. wilfordii***	Cynawilfoside A, cynauricoside A, wilfoside C1N, wilfoside K1N and cyanoauriculoside G	Roots	In vivo	Retigabine: 15.0 mg/kg	Model: MES-induced mouse seizure model. ED_50_ values of 48.5, 95.3, 124.1, 72.3, and 88.1 mg/kg, respectively.	[50]
***C. otophyllum***	Otophylloside B	Roots	In vivo	Curcumin: 100 μM	Model: AD (Alzheimer’s disease). 50 μM	[106]
**Antifungal ,parasitic and antiviral Activity**						
***C. wilfordii***	Wilfoside C1N, wilfoside C1G, wilfoside C1GG	Roots	In vivo	PolyoxinB (IC_50_ value = 71.36 μg/mL)	Model: Barley powdery mildew. The IC_50_ (i.e., the concentration required for 50% inhibition) were determined as 3.24 μg/mL, 12.90 μg/mL, and 28.35 μg/mL, respectively.	[27]
***C. paniculatum***	Ethyl acetate (EA) extracts	Roots	In vitro	Amantadine	Model: Madin-Darby bovine kidney (MDBK) cells. The tissue culture infectious dose assay (TCID_50_) assay. The cytotoxic concentration CC_50_ was 18.2 μg/mL; The EA MNTD (Maximum non-toxic dose) is 18.2 μg/mL.	[107]
***C. atratum***	Cynatratoside C	Roots	In vitro		Model: Grasscarp infected with *I. multifiliis.* 0.25 mg/L.	[10]
***C. paniculatum***	Cynatratoside A; cynanversicoside C	Roots	In vitro		Cynatratoside A and cynanversicoside C could be 100% effective against *I. multifiliis* at the concentration of 10.0 mg L^−1^, with the median effective concentration (EC_50_) values of 4.6 and 5.2 mgL^−1^, respectively.	[11]
***C. paniculatum***	Essential oil	Roots	In vitro	Benzyl benzoate and DEET (diethylmethylbenzamide) 1.13 μg/cm^2^	LD_50_ were 8.93, 4.58, and 2.79. It showed more toxic than DEET (LD_50_ = 4.13, 3.91, and 4.87 μg/cm^2^) against D. farinae, D. pteronyssinus, and T. putrescentiae, respectively.	[108]
***C. komarovii***	7-demethoxytylophorine(1),7-demethoxytylophorine *N*-oxide(2)	Roots	In vitro	2,4-dioxo-hexahydro-1,3,5-triazine, showed 50% inhibition at 500 μg/mL	The alkaloid 1 exhibited 65% inhibition against the TMV at a concentration of 1.0 μg/mL. Alkaloid 2 showed 60% inhibition at 500 μg/mL	[92]
***C. atratum***	Cynanoside A,G,M; glaucogenin-C 3-*O*-*β*-d-cymaropyranosyl-(1→4)-*α*-L-diginopyranosyl-(1→4)-*β*-d-cymaropyranoside; glaucogenin-A 3-*O*-*β*-d-cymaropyranosyl-(1→4)-*α*-L-diginopyranosyl-(1→4)-*β*-d-cymaropyranoside	Roots	In vivo	Ningnanmycin (IC_50_ = 49.6 μg/mL).	IC_50_ = 20.5 μg/mL, IC_50_ = 18.6 μg/mL, IC_50_ = 22.0 μg/mL, IC_50_ = 19.2 μg/mL, IC_50_ = 22.2 μg/mL, respectively.	[36]
***C. stauntonii***	Volatile oil	Roots	In vitro	300 mg/kg 6 d	Model: Mouse influenza model. IC_50_ = 64 μg/mL	[109]
**Immunosuppressive activity**						
***C. chekiangense***	Chekiangensosides A, cynajapogenin A, chekiangensoside B, glaucogenin A	Roots	In vitro	cyclosporin A	Model: Con A- and LPS-induced proliferation of mice splenocytes. 100 μL (0.01–10 g/mL)	[23]
***C. atratum***	Atratcynoside A, atratcynoside B, atratcynoside C	Roots	In vitro	Cyclosporin A: 0.09 ± 0.01 μM	Model: Con A-induced proliferation of T-lymphocytes from mice. IC_50_ values of 3.3 μM, 7.0 μM, 6.7 μM, respectively.	[72]
**Anti-inflammatory activity**						
***C. stauntonii***	Cynastauoside B; cynastauoside C	Roots	In vitro	Dexamethasone with the inhibition ratio of 83.5% at a concentration of 1 μM.	Model: C57bl/6j mouse peritoneal macrophages. The results showed 17.0% and 6.9% of inhibition rate at a concentration of 10 μM, respectively.	[74]
***C. wilfordii.***	Cynandione A	Roots	In vitro		Model: LPS-Induced BV-2 microglial cells. IC_50_ = 27.13 ± 5.38 μM.	[110]
***C. stauntonii***	Stauntoside V1; stauntoside V3	Roots	In vitro	Dexamethasone: IC_50_ = 0.3 μM	Model: C57bl/6j mouse peritoneal macrophages. IC_50_ values of 9.3 μM and 12.4 μM, respectively.	[111]
***C. atratum***	Aqueous extract	Roots	In vivo	dexamethasone	Model: Female BALB/c mice/atopic Dermatitis (AD) and Human mast cell line (HMC-1). 1 or 100 mg/mL.	[112]
			In vitro			
***C. wilfordii***	Polysaccharides	Roots	In vivo	5-aminosalicylic acid (100 mg/kg)	Model: DSS (dextran sodium sulfate)-induced chroniccolitis in mice. 200 mg/kg or 100 mg/kg	[113]
			In vitro		Model: LPS-induced RAW 264.7 macrophages. 25 μg/mL	
**Anti-oxidation**						
***C. wilfordi***	Gagaminine	Roots	In vivo	Pyridoxal: IC_50_ = 246 μM	Model: Rat liver injury model. IC_50_ = 0.8 μM (0.5 μg/mL)	[94]
***C. otophyllum***	Otophyllosides A and B	Roots	In vivo		These compounds could protect rats from audiogenic seizures and ED_50_ value of 10.2 mg/kg.	[8]
**Hepatoprotective activity**						
***C. wilfordii ***	Cynandione A	Roots	In vitro	Silybin (100 μM)	Model: Primary cultures of rat hepatocytes injured by CCl_4_. 50 μM	[114]
***C. wilfordii ***	Crude extract (CWE)	Roots	In vivo	Simvastatin/10 mg/kg/day/12 weeks CWE:100 and 200 mg/kg/day/12 weeks	Model: Male C57BL/6 mice. CWE can inhibit fat accumulation in the liver. Suppressing lipid accumulation in the liver and reducing blood levels of total cholesterol and triglycerides.	[115]
**Appetite suppressant effect**						
***C.auriculatum***	Wilfoside K1N	Roots	In vivo	Sibutramine 15 mg/kg body weight Compound: 50 mg/kg body weight	Model: SPF female Wistar rats.	[30]
**Antidepressant activity**						
***C. auriculatum***	Cynanauriculoside C, cynanauriculoside D, cynanauriculoside E, otophylloside L, cynauricuoside C	Roots	In vivo	fluoxetine (20 mg/kg) Compound: 50 mg/kg (i.g.)/twice a day/5 d Male ICR mice (18–22 g)	These compounds could significant antidepressant activity at the dosage of 50 mg/kg (i.g.)	[67]
**Vasodilating activity**						
***C. stauntonii***	Stauntonine	Roots	In vivo		IC_50_ = 5.37 × 10^−6^ mol/L	[40]
***C. auriculatum***	Caudatin		In vitro and In vivo		Model: HUVEC human umbilical vein endothelial cell and U251 human glioma cells xenograft model. 25–200 μM.	[116]
**Others**						
***C. bungei***	2,5-dihydroxyacetophenone (2,5-DHAP)	Roots	In vitro and In vivo	Standard depigmenting agent: 0.2 mM	0.4 mM	[79]
***C. stauntonii***	Stauntosaponins A and B	Roots	In vitro	Ouabain: IC_50_ value of 3.5 μM. Assay of Na+/K+-ATPase inhibition	IC_50_ = 21 μM and IC_50_ = 29 μM	[77]
***C. taiwanianum***	Cynandione B	Plants	In vitro		Model: The formyl-methionyl-leucyl-phenylalanine (fMLP)-stimulated rat neutrophil washed rabbit platelets induced by arachidonic acid. IC_50_ = 1.5 ± 0.2 and 1.6 ± 0.2 μM, respectively.	[117]
	2,5-Dihydroxyacetophenone				IC_50_ = 4.8 μM.	
***C. stauntonii***	Cynatratoside B	Roots	In vitro	Isoprenaline: IC_50_ = 0.13 μM	Model: Rat Tracheal Rings Preparation. The EC_50_ acetylcholine- and carbachol-induced contraction of compound were 0.67 and 0.38 μg/mL (∼0.85 and 0.48 μM), respectively.	[12]
